# A Pathogen and a Non-pathogen Spotted Fever Group *Rickettsia* Trigger Differential Proteome Signatures in Macrophages

**DOI:** 10.3389/fcimb.2019.00043

**Published:** 2019-03-06

**Authors:** Pedro Curto, Cátia Santa, Paige Allen, Bruno Manadas, Isaura Simões, Juan J. Martinez

**Affiliations:** ^1^PhD Programme in Experimental Biology and Biomedicine, Center for Neuroscience and Cell Biology, University of Coimbra, Coimbra, Portugal; ^2^Institute for Interdisciplinary Research, University of Coimbra, Coimbra, Portugal; ^3^CNC-Center for Neuroscience and Cell Biology, University of Coimbra, Coimbra, Portugal; ^4^Vector Borne Disease Laboratories, Department of Pathobiological Sciences, LSU School of Veterinary Medicine, Baton Rouge, LA, United States

**Keywords:** *Rickettsia conorii*, *Rickettsia montanensis*, spotted fever group *Rickettsia*, macrophages, SWATH-MS, host-pathogen interactions, metabolic reprogramming, protein processing pathways

## Abstract

We have previously reported that *Rickettsia conorii* and *Rickettsia montanensis* have distinct intracellular fates within THP-1 macrophages, suggesting that the ability to proliferate within macrophages may be a distinguishable factor between pathogenic and non-pathogenic Spotted fever group (SFG) members. To start unraveling the molecular mechanisms underlying the capacity (or not) of SFG *Rickettsia* to establish their replicative niche in macrophages, we have herein used quantitative proteomics by SWATH-MS to profile the alterations resulted by the challenge of THP-1 macrophages with *R. conorii* and *R. montanensis*. We show that the pathogenic, *R. conorii*, and the non-pathogenic, *R. montanensis*, member of SFG *Rickettsia* trigger differential proteomic signatures in macrophage-like cells upon infection. *R. conorii* specifically induced the accumulation of several enzymes of the tricarboxylic acid cycle, oxidative phosphorylation, fatty acid **β**-oxidation, and glutaminolysis, as well as of several inner and outer membrane mitochondrial transporters. These results suggest a profound metabolic rewriting of macrophages by *R. conorii* toward a metabolic signature of an M2-like, anti-inflammatory activation program. Moreover, several subunits forming the proteasome and immunoproteasome are found in lower abundance upon infection with both rickettsial species, which may help bacteria to escape immune surveillance. *R. conorii*-infection specifically induced the accumulation of several host proteins implicated in protein processing and quality control in ER, suggesting that this pathogenic *Rickettsia* may be able to increase the ER protein folding capacity. This work reveals novel aspects of macrophage-*Rickettsia* interactions, expanding our knowledge of how pathogenic rickettsiae explore host cells to their advantage.

## Introduction

Bacteria in the genus *Rickettsia* are small Gram-negative α-proteobacteria, which can be transmitted to humans through arthropod vectors (Hackstadt, 1996). Although rickettsial species share a high degree of genome similarity, they are associated with very different clinical outcomes (Fang et al., 2017), and the molecular determinants underlying these drastic differences in pathogenicity between *Rickettsia* species are still to be understood.

Endothelial cells have long been considered the primary target cells for *Rickettsia* (Walker and Ismail, 2008). However, even pathogens that preferentially invade non-macrophage cells might encounter macrophages during their experience in the extracellular space or when the primary host cell undergoes apoptosis, and subsequent phagocytosis by a nearby macrophage (Walker and Gear, 1985; Walker, 1997; Price and Vance, 2014). New evidence of the presence of intact *Rickettsia* within the cytoplasm of macrophages, both in tissues and within the blood circulation, has raised further questions about the exact role of these phagocytic cells in the pathogenesis of rickettsial diseases (Walker and Gear, 1985; Banajee et al., 2015; Riley et al., 2016). Over 40 years ago, it was shown that two *Rickettsia* strains of the Typhus Group with different levels of virulence displayed distinct capacities to proliferate within macrophages (Gambrill and Wisseman, 1973). More recently, we have reported that *R. conorii*, the causative agent of Mediterranean spotted fever (MSF) and *R. montanensis*, a genetically related species not associated with disease in humans, differ in their ability to proliferate within THP-1 macrophages (Curto et al., 2016). Combined, these results are suggestive of an association between the ability to replicate in macrophages and rickettsial pathogenicity, which may help to explain why certain species of *Rickettsia* are not associated with disease. However, *Rickettsia*-macrophage interactions are still very poorly understood.

It is known that many pathogenic bacteria have evolved sophisticated strategies to escape macrophage immune defenses, being able to replicate within these as well as in other phagocytic cells (Sarantis and Grinstein, 2012; Price and Vance, 2014). For many intracellular bacteria, replication and/or survival within macrophages has been related with the ability to cause disease (Price and Vance, 2014). The diversity of functions that can be performed by macrophages is directly linked to a high degree of metabolic diversity and plasticity as well as a fast ability to respond to specific environments (Martinez and Gordon, 2014; Murray et al., 2014; Price and Vance, 2014). These features are considered very attractive to be explored by intracellular pathogens as a vast source of cellular resources that can be rapidly remodeled (Eisenreich et al., 2015, 2017; Van den Bossche et al., 2017). Along with the capacity to hijack a wide range of host signaling pathways to their own benefit (Ashida et al., 2014; Reddick and Alto, 2014; Friedrich et al., 2017), it has been reported that several intracellular pathogens are also able to induce distinct host cell metabolic signatures in macrophages to suit their replication requirements (Eisele et al., 2013; Xavier et al., 2013). The altered metabolic state of M2 macrophages, which has also been associated with reduced antimicrobial capacity, seems to be a beneficial factor that supports the survival and proliferation of several intracellular pathogens (Benoit et al., 2008; Mege et al., 2011; Eisele et al., 2013; Buchacher et al., 2015). Overall, intracellular bacteria appear to be able to capitalize on macrophage intrinsic plasticity for optimal replication during infection (Price and Vance, 2014). This is particularly relevant in *Rickettsia* since reductive genome evolution has resulted in the purge of many metabolic pathways in these obligate intracellular bacteria, resulting in a strict dependency on the host cell to replicate (Driscoll et al., 2017).

The drastic intracellular phenotypic differences between *R. conorii* and *R. montanensis* in THP-1 macrophages (Curto et al., 2016), suggest substantial alterations in the content of host proteins, which may likely reflect differential macrophage responses to either favor (*R. conorii*) or restrict (*R. montanensis*) intracellular bacterial proliferation. To gain deeper insights into these host responses, we herein employed a label-free quantitative proteomics approach (SWATH-MS) (sequential window acquisition of all theoretical mass spectra), to profile proteomic alterations that occur upon infection of THP-1 macrophages with *R. conorii* and *R. montanensis*. SWATH-MS is a highly specific data-independent acquisition method that has been successfully used to compare alterations in protein content in different experimental contexts (Anjo et al., 2017; Gao et al., 2017). Our results revealed substantial differences in protein content between infection conditions, with two main targeted modules, carbon metabolism, and protein processing pathways, emerging as differentially affected upon infection with each rickettsial species. Differential changes observed in proteins associated with key metabolic pathways anticipate the induction of distinct metabolic signatures by *R. conorii* and *R. montanensis*. Moreover, our results revealed a reduced abundance of different proteasome and immunoproteasome subunits upon infection with both rickettsial species, while *R. conorii*-infection specifically induced the accumulation of different ER quality control proteins. Overall, our results point toward a substantial manipulation of the host by the pathogen *R. conorii* to meet host cell bioenergetics demands and sustain cell viability for bacterial replication, and, likely, to maintain its own metabolic needs.

## Materials and Methods

### Cell Lines, *Rickettsia* Growth, and Purification

Vero cells were grown in Dulbecco's modified Eagle's medium (DMEM; Gibco) supplemented with 10% heat-inactivated fetal bovine serum (Atlanta Biologicals), 1x non-essential amino acids (Corning), and 0.5 mM sodium pyruvate (Corning). THP-1 (ATCC TIB-202^TM^) cells were grown in RPMI-1640 medium (Gibco) supplemented with 10% heat-inactivated fetal bovine serum (Atlanta Biologicals). Differentiation of THP-1 cells into macrophage-like cells was carried out by the addition of 100 nM of phorbol 12-myristate 13-acetate (PMA; Fisher). Cells were allowed to differentiate and adhere for 3 days prior to infection. Both cell lines were maintained in a humidified 5% CO_2_ incubator at 34°C. *R. conorii* isolate Malish7 and *R. montanensis* isolate M5/6 were propagated in Vero cells and purified as previously described (Ammerman et al., 2008; Chan et al., 2009; Chan et al., 2011).

### Sample Preparation

PMA-differentiated THP-1 cells monolayers at a cell confluency of 2 × 10^5^ cells per well, in 24 well plates (3 wells per condition) were infected with *R. conorii, R. montanensis* at a multiplicity of infection (MOI) of 10 or maintained uninfected. Plates were centrifuged at 300 x g for 5 min at room temperature to induce contact between rickettsiae and host cells, and incubated at 34°C and 5% CO_2_ for 24 h. At the specified time point, culture medium was removed, cells were washed 1x with PBS and total protein was extracted using 100 μL of protein extraction buffer per well [25 mM Tris/HCl, 5 mM EDTA, 1% Triton X-100, and Pierce protease inhibitors table (ThermoFisher Scientific), pH 7.0]. Samples were passed 10 times through Insulin Syringe with 28-gauge needle (Becton Dickinson) and denatured using 6x SDS sample buffer (4x Tris/HCl, 30% glycerol, 10% SDS, 0.6 M DTT, 0.012% Bromophenol Blue, pH 6.8) during 10 min at 95°C. Total protein content in each sample was then quantified using 2D Quant kit (GE Healthcare) and kept at −20°C until further processing. Experiments were done in quadruplicate. After thawing, 10 μg of each replicate sample from each experimental condition were pooled together, creating this way three pooled samples (*R. conorii* pool, *R. montanensis* pool, and uninfected pool). At this point, the same amount of a recombinant protein [Green fluorescent Protein and Maltose-binding periplasmic protein (malE-GFP)] was added to each replicate sample and pooled samples to serve as an internal standard. All the samples were boiled for 5 min and acrylamide was added as an alkylating agent.

### In-gel Digestion and Liquid Chromatography Coupled to Tandem Mass Spectrometry (LC-MS/MS)

The volume corresponding to 40 μg of each replicate sample as well as pooled samples was then loaded into a precast gel (4–20% Mini-Protean® TGX™ Gel, Bio-Rad), and the SDS-PAGE was partially run for 15 min at 110 V (Anjo et al., 2015). After SDS-PAGE, proteins were stained with Colloidal Coomassie Blue as previously described (Manadas et al., 2009).

The lanes were sliced into 3 fractions with the help of a scalpel, and after the excision of the gel bands, each one was sliced into smaller pieces. The gel pieces were destained using a 50 mM ammonium bicarbonate solution with 30% acetonitrile (ACN) followed by a washing step with water [each step was performed in a thermomixer (Eppendorf) at 1,050 x rpm for 15 min]. The gel pieces were dehydrated on Concentrator Plus/Vacufuge® Plus (Eppendorf). To each gel band 75 μL of trypsin (0.01 μg/μL solution in 10 mM ammonium bicarbonate) were added to the dried gel bands and left for 15 min at 4°C to rehydrate the gel pieces. After this period, 75 μL of 10 mM ammonium bicarbonate were added and in-gel digestion was performed overnight at room temperature in the dark. After digestion, the excess solution from gel pieces was collected to a low binding microcentrifuge tube (LoBind®, Eppendorf) and peptides were extracted from the gel pieces by sequential addition of three solutions of increasing percentage of acetonitrile (30, 50, and 98%) in 1% formic acid (FA). After the addition of each solution, the gel pieces were shaken in a thermomixer (Eppendorf) at 1,250 rpm for 15 min and the solution was collected to the tube containing the previous fraction. The peptide mixtures were dried by rotary evaporation under vacuum (Concentrator Plus/Vacufuge® Plus, Eppendorf). The peptides from each fraction of each sample were pooled together for SWATH analysis; the peptides from the pooled samples were kept separated in the three fractions of the digestion procedure.

After digestion, all samples were subjected to solid phase extraction with C18 sorbent (OMIX tip, Agilent Technologies). The eluted peptides were evaporated and solubilized in 30 μL mobile phase, aided by ultrasonication using a cuphorn device (Vibra-cell 750 watt, Sonics) at 40% amplitude for 2 min. Samples were then centrifuged for 5 min at 14,100 × g (minispin plus, Eppendorf) and analyzed by LC-MS/MS.

The Triple TOF™ 5600 System (Sciex) was operated in two phases: information-dependent acquisition (IDA) of each fraction of the pooled samples; followed by SWATH (Sequential Windowed data independent Acquisition of the Total High-resolution Mass Spectra) acquisition of each sample. Peptide separation was performed using liquid chromatography (nanoLC Ultra 2D, Eksigent) on a ChromXP C18CL reverse phase column (300 μm × 15 cm, 3 μm, 120 Å, Eksigent) at 5 μL/min with a 45 min gradient from 2 to 35% acetonitrile in 0.1% FA, and the peptides were eluted into the mass spectrometer using an electrospray ionization source (DuoSpray™ Source, Sciex).

Information dependent acquisition (IDA) experiments were performed by analyzing 10 μL of each fraction of the pooled samples. The mass spectrometer was set for IDA scanning full spectra (350–1,250 m/z) for 250 ms, followed by up to 100 MS/MS scans [100–1,500 m/z from a dynamic accumulation time—minimum 30 ms for precursor above the intensity threshold of 1,000 counts per second (cps)—in order to maintain a cycle time of 3.3 s]. Candidate ions with a charge state between +2 and +5 and counts above a minimum threshold of 10 cps were isolated for fragmentation and one MS/MS spectra was collected before adding those ions to the exclusion list for 25 s (mass spectrometer operated by Analyst® TF 1.7, Sciex). Rolling collision energy was used with a collision energy spread of 5.

The SWATH setup was essentially as in Gillet et al. (2012), with the same chromatographic conditions used for SWATH and IDA acquisitions. For SWATH-MS based experiments, the mass spectrometer was operated in a looped product ion mode. The SWATH-MS setup was designed specifically for the samples to be analyzed (Supplementary Table 1), in order to adapt the SWATH windows to the complexity of this batch of samples. A set of 60 windows of variable width (containing 1 m/z for window overlap) was constructed covering the precursor mass range of 350–1,250 m/z. A 200 ms survey scan (350–1,250 m/z) was acquired at the beginning of each cycle for instrument calibration and SWATH MS/MS spectra were collected from 100 to 1,500 m/z for 50 ms resulting in a cycle time of 3.25 s from the precursors ranging from 350 to 1,250 m/z. The collision energy for each window was determined according to the calculation for a charge +2 ion centered upon the window with variable collision energy spread (CES) according with the window.

### Protein Identification and Relative Quantification

Specific library of precursor masses and fragment ions were created by combining all files from the IDA experiments, and used for subsequent SWATH processing. The library was obtained using ProteinPilot™ software (v5.0.1, Sciex), with the following search parameters: *Homo Sapiens* SwissProt database (release of March 2017) and malE-GFP; acrylamide alkylated cysteines as fixed modification; and the gel based special focus option. An independent False Discovery Rate (FDR) analysis using the target-decoy approach provided with ProteinPilot™ software was used to assess the quality of the identifications, and positive identifications were considered when identified proteins and peptides reached a 5% local FDR (Tang et al., 2008; Sennels et al., 2009).

Data processing was performed using SWATH™ processing plug-in for PeakView™ (v2.2, Sciex), briefly peptides were selected from the library using the following criteria: (i) the unique peptides for a specific targeted protein were ranked by the intensity of the precursor ion from the IDA analysis as estimated by the ProteinPilot™ software, and (ii) Peptides that contained biological modifications and/or were shared between different protein entries/isoforms were excluded from selection. Up to 15 peptides were chosen per protein, and SWATH™ quantitation was attempted for all proteins in the library file that were identified below 5% local FDR from ProteinPilot™ searches. Peptide's retention time was adjusted by using the malE-GFP peptides. In SWATH™ Acquisition data, peptides are confirmed by finding and scoring peak groups, which are a set of fragment ions for the peptide. Up to 5 target fragment ions were automatically selected and the peak groups were scored following the criteria described in Lambert et al. (2013). Peak group confidence threshold was determined based on a FDR analysis using the target-decoy approach and 1% extraction FDR threshold was used for all the analyses. Peptides that met the 1% FDR threshold in at least one pair of technical replicates were retained, and the peak areas of the target fragment ions of those peptides were extracted across the experiments using an extracted-ion chromatogram (XIC) window of 4 min. Protein levels were estimated by summing all the transitions from all the peptides for a given protein (Collins et al., 2013) and normalized to the total intensity at the protein level. Statistical tests were performed in SPSS (v23, IBM) using the non-parametric Mann Whitney *U*-test and proteins were considered altered when an alteration of at least 20% in abundance (fold change ≤ 0.83 or fold change ≥ 1.2) was observed between uninfected and infected conditions.

### Bioinformatics Analysis

The correlation plots of the quantitative data from all the proteins quantified were obtained using InfernoRDN (v1.1) software (Polpitiya et al., 2008). Principal components analysis was performed using the software MakerView (v1.2.1, Sciex). The analysis was attempted by importing the quantitative data from the proteins considered as altered upon infection of THP-1 macrophages with *R. conorii* or *R. montanensis*. Functional protein association networks were evaluated using the Search Tool for Retrieval of Interacting Genes/Proteins (STRING) 10.5 (http://string-db.org/) with high confidence (0.7) parameters (Szklarczyk et al., 2017). Quantified proteins that were considered significantly differentially represented in each experimental condition were also upload into KEGG pathway databases (http://www.genome.jp/kegg/pathway.html) to identify significant altered canonical pathways (Kanehisa et al., 2017).

### Pharmacological Inhibition of FASN

THP-1 cells were differentiated in 96 well plates at 5 × 10^4^ cell per well in a total volume of 200 μL. Three days after addition of PMA (Fisher), differentiated THP-1 cells were infected in triplicate for each indicated time point with *R. conorii* Malish7 at a MOI of 2 and centrifuged at 300 × g for 5 min to induce contact. Cerulenin (Sigma-Aldrich) was added at a final concentration of 0.03 mM, while control cells were treated with an equal volume of DMSO. Cerulenin-treated and control cells that were infected were harvested by scraping at 24 h, 3 days, and 5 days post-treatment and stored in 1 x PBS at −80°C. Samples were individually processed for total gDNA isolation and subsequent quantitative PCR analysis for quantification of rickettsiae as previously described (Curto et al., 2016).

### Silencing of *FASN* Using siRNA

The Viromer Blue siRNA transfection reagent (Lipocalyx) was used to silence the expression of *FASN*, using manufacturer's suggested protocol with slight modifications. Briefly, THP-1 cells were PMA-differentiated for 24 h in 96 well plates at 5 × 10^4^ cells per well in 200 μL of media. Small interfering RNA (siRNA) targeting fatty acid synthase (siFASN; Ambion) or a scrambled RNA used as a control (siIRR; Ambion) were added to differentiated THP-1 cells at a final concentration of 2.75 μM. After 24 h of transfection, siRNA treated THP-1 cells were infected in triplicate for each indicated time point with *R. conorii* Malish7 at a MOI of 2 and centrifuged at 300 x g for 5 min to induce contact. Infected cells were harvested by scraping at 24 h, 3 days, and 5 days post infection and stored in 1x PBS at −80°C until gDNA extractions and quantitative PCR was conducted as described previously (Curto et al., 2016). To determine the efficacy of the RNA silencing on protein expression, total protein from transfected THP-1 cells at 3 days post-infection was extracted in 25 μL per well of 1% NP-40 lysis buffer as previously described (Riley et al., 2014) and then boiled in 6x SDS-PAGE loading buffer. Total protein content was normalized using the 2-D Quant Kit (GE Healthcare) and then equal protein content was separated on 4–20% gradient SDS-PAGE before being transferred to nitrocellulose. Proteins of interest were detected by chemiluminescent Western blotting and exposure to film using the following antibodies: anti-FASN (Abcam) and anti-β-actin (Sigma) followed by Donkey anti-Rabbit-HRP (Sigma) and Donkey anti-Mouse-HRP (Sigma).

### Western Blotting

After thawing, the same amount of protein for each sample was resolved by SDS-PAGE using 12.5% polyacrylamide gels in a Bio-Rad Mini-Protean Tetra Cell and transferred to a polyvinylidene difluoride membrane at 100 V during 90 min at 4°C. The membranes were blocked for 60 min with 5% non-fat dry milk in Tris-buffered saline (TBS) containing 0.1% Tween 20 and then incubated at 4°C overnight with primary antibodies. The following primary antibodies were used accordingly: anti-GAPDH (0411) (Santa Cruz Biotechnology, sc-47724) (1:200); Membrane Integrity WB Antibody Cocktail (Abcam, ab110414) (1:1,000) for detection of VDAC1 and cytochrome c; anti-PSMA2 (Cell Signaling Technology, #2455) (1:1,000); Total OXPHOS Human WB Antibody Cocktail (Abcam, ab110411) (1:1,000) for detection of UQCR2, SDHB, COX II, and NDUFB8; monoclonal anti-β-actin (clone AC-15) (Sigma, A5441) (1:5,000). After several washes with 0.5% non-fat dry milk plus 0.1% Tween 20 in TBS, the membranes were incubated at room temperature with the correspondent secondary antibodies: anti-Mouse IgG (whole molecule)-peroxidase antibody produced in rabbit (Sigma, A9044) (1:10,000) and anti-rabbit IgG alkaline phosphatase linked whole antibody produced in goat (GE Healthcare, NIF1317) (1:20,000). The membranes were washed again in TBS containing 0.1% Tween 20, and visualized by the enhanced chemiofluorescence method using ECF substrate (GE Healthcare) on a Molecular Imager FX System (Bio-Rad) or using NZY Supreme ECL HRP substrate (NZYTech) on a VWR® Imager (VWR), depending on the secondary antibody used.

## Results

### Global Changes in Proteome Profiles Stimulated by *R. conorii* and *R. montanensis* Infection in THP-1 Macrophages

**Figure 1:**
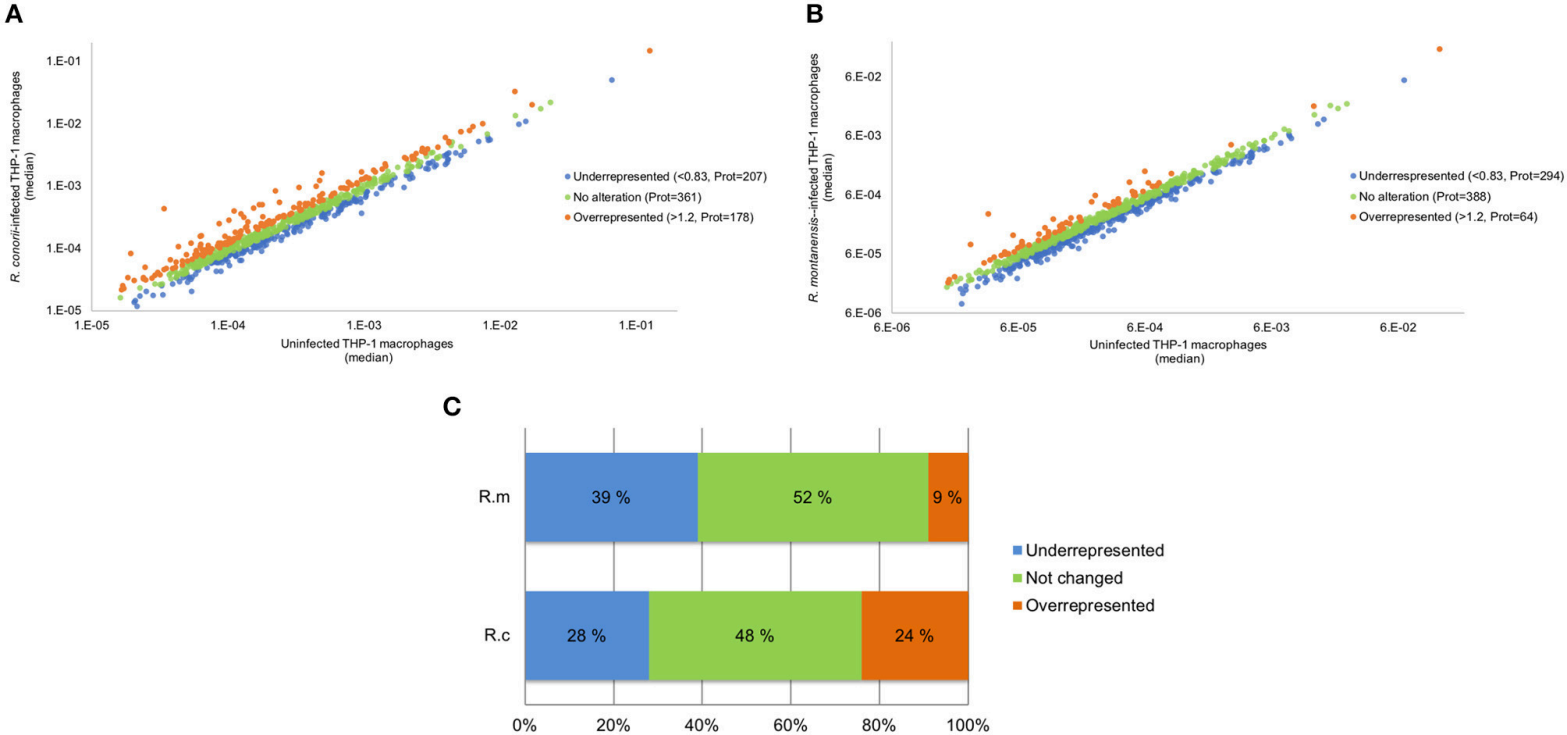
Overall analysis of *R. conorii*- and *R. montanensis*-induced changes in global proteome of THP-1 macrophages. **(A,B)** Scatterplot representation of changes in protein abundance of THP-1 macrophages upon infection with *R. conorii* **(A)** and *R. montanensis* **(B)**. The 746 quantified proteins that were confidently quantified in all 3 experimental conditions were plotted and considered altered when a change of at least 20% in abundance (fold change ≤ 0.83 or fold change ≥ 1.2) was observed between infected and uninfected conditions. Proteins that were considered to decrease, not change or increase its abundance upon infection are represented in blue, green and orange, respectively. **(C)** Bar chart displaying the percentage (out of the 746 quantified proteins that were confidently quantified) of host proteins that were considered to decrease, not change or increase its abundance upon infection with both *R. conorii* and *R. montanensis*. See also Supplementary Table 2.

We have previously shown that at 24 h post-infection (hpi), pathogenic *R. conorii* are present as intact bacteria and free in the cytoplasm in macrophage-like cells, whereas non-pathogenic *R. montanensis* are rapidly destroyed, with rickettsial debris showing substantial co-localization with lysosomal markers (Curto et al., 2016). To gain insights into molecular changes associated with these opposite phenotypes, we compared host protein abundance in infected and uninfected cells using a label-free quantitative proteomics approach. The use of *R. montanensis*-infected cells as an additional experimental condition allows to establish the typical responses of THP-1 macrophages to clear an infection vs. those induced by the pathogen *R. conorii* that proliferates in these cells. Total protein extracts were prepared from PMA-differentiated THP-1 cells at 24 h post-infection with *R. conorii* and *R. montanensis* (MOI = 10), and from uninfected cells processed in parallel. The relative protein quantification was performed using LC-SWATH-MS analysis, where a comprehensive library of 1,425 confidently identified proteins was created and, from which, a total of 746 proteins were confidently quantified in all samples. Proteins were considered as altered when an alteration of at least 20% in abundance (fold change ≤ 0.83 or fold change ≥ 1.2) was observed between uninfected and infected conditions (Rukmangadachar et al., 2016; Bussey et al., 2018). Using these criteria, THP-1 macrophages infected with *R. conorii* showed significant changes in the content of a total of 385 proteins compared to uninfected cells. Of these, 178 (corresponding to 24% of the quantified proteins) were found enriched, while 207 (28% of the quantified proteins) showed reduced abundance (Figures 1A,C and Supplementary Table 2). On the other hand, in THP-1 macrophages infected with *R. montanensis*, we identified a total of 358 proteins with significantly altered abundance, 64 (9% of the quantified proteins) of which identified as enriched and 294 (39% of the quantified proteins) with reduced abundance compared to uninfected cells (Figures 1B,C and Supplementary Table 2). Principal component analysis (PCA) was carried out to assess the sample correlations using the quantification data of altered proteins upon infection of THP-1 macrophages with *R. conorii* or *R. montanensis* (Supplementary Figure 1).

To provide insights on cellular pathways associated with these significantly altered proteins, we performed a KEGG pathway enrichment analysis (Kanehisa et al., 2017) using STRING databases (von Mering et al., 2003; Szklarczyk et al., 2017). Top pathways enriched among over and underrepresented proteins in *R. conorii* and *R. montanensis*-infected cells are shown in Tables 1, 2, respectively. In *R. conorii*-infected cells, several proteins with either increased and reduced abundance were categorized in broad term categories such as metabolic pathways (KEGG:1100) and carbon metabolism (KEGG:1200), suggesting a significant impact of infection in different pathways of host metabolism, as dissected in detail below. Moreover, accumulating proteins were also associated with protein processing in the endoplasmic reticulum (KEGG:4141), while proteins with reduced abundance were associated with proteasome (KEGG:3050). In *R. montanensis*-infected cells, the observed pathway enrichment pattern is different, with top pathways among underrepresented proteins related with proteasome (KEGG:3050), spliceosome (KEGG:3040), carbon metabolism (KEGG:1200), and metabolic pathways (KEGG:1100), whereas in the group of enriched proteins, the top pathways were related to ribosome (KEGG:3010) and complement and coagulation cascades (KEGG:4610) (although with fewer proteins associated).

**Table 1:** KEGG pathways enriched among under and overrepresented proteins upon infection of THP-1 macrophages with *R. conorii*.

**ID**	**Pathway description**	**Count in gene set**	**False discovery rate**
**KEGG Pathways**			
***R. conorii-*****Infected Cells—Proteins With Reduced Abundance**			
1200	Carbon metabolism	13	8.61E-09
1100	Metabolic pathways	38	1.26E-08
30	Pentose phosphate pathway	7	4.90E-07
10	Glycolysis/Gluconeogenesis	9	5.46E-07
3050	Proteasome	8	5.46E-07
1230	Biosynthesis of amino acids	9	1.57E-06
5130	Pathogenic *Escherichia coli* infection	8	1.88E-06
5203	Viral carcinogenesis	12	1.08E-05
480	Glutathione metabolism	7	1.76E-05
***R. conorii-*****Infected Cells—Proteins With Enriched Abundance**			
4141	Protein processing in endoplasmic reticulum	23	3.69E-19
5012	Parkinson's disease	16	9.80E-12
1100	Metabolic pathways	36	1.40E-09
5016	Huntington s disease	15	3.68E-09
20	Citrate cycle (TCA cycle)	8	3.72E-09
1200	Carbon metabolism	12	3.72E-09
5010	Alzheimer s disease	12	7.80E-07
510	N-Glycan biosynthesis	7	6.07E-06
4260	Cardiac muscle contraction	8	8.43E-06
3060	Protein export	5	2.99E-05
190	Oxidative phosphorylation	9	4.95E-05

**Table 2:** KEGG pathways enriched among under and overrepresented proteins upon infection of THP-1 macrophages with *R. montanensis*.

**ID**	**Pathway description**	**Count in gene set**	**False discovery rate**
**KEGG PATHWAYS**			
***R. montanensis*****-Infected Cells—Proteins With Reduced Abundance**			
3050	Proteasome	15	4.41E-15
3040	Spliceosome	19	2.06E-12
1200	Carbon metabolism	16	1.19E-10
1100	Metabolic pathways	48	1.90E-09
10	Glycolysis/Gluconeogenesis	11	4.39E-08
30	Pentose phosphate pathway	8	9.36E-08
5130	Pathogenic *Escherichia coli* infection	9	1.92E-06
1230	Biosynthesis of amino acids	10	2.01E-06
5203	Viral carcinogenesis	14	1.12E-05
480	Glutathione metabolism	8	1.22E-05
5169	Epstein-Barr virus infection	14	1.55E-05
620	Pyruvate metabolism	7	3.35E-05
4114	Oocyte meiosis	10	5.81E-05
***R. montanensis*****-Infected Cells—Proteins With Enriched Abundance**			
3010	Ribosome	5	0.00773
4610	Complement and coagulation cascades	4	0.00773
5143	African trypanosomiasis	3	0.013
510	N-Glycan biosynthesis	3	0.0238
5144	Malaria	3	0.0238
5150	*Staphylococcus aureus* infection	3	0.0238
5012	Parkinson's disease	4	0.0344
4141	Protein processing in endoplasmic reticulum	4	0.044
4260	Cardiac muscle contraction	3	0.044
4964	Proximal tubule bicarbonate reclamation	2	0.044

**Figure 2:**
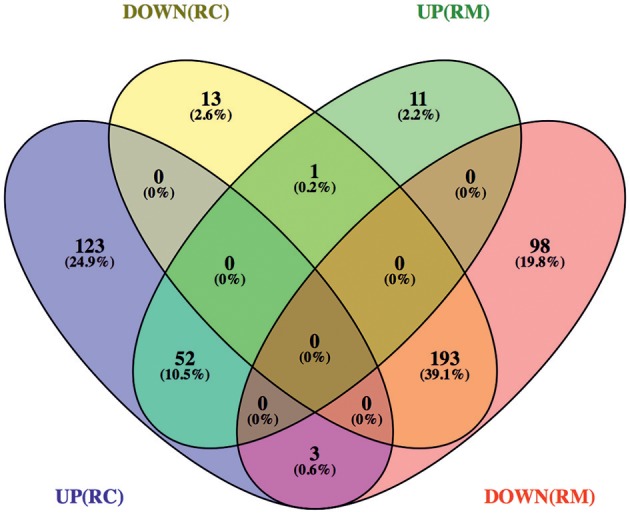
Venn diagram depicting the number and distribution of host proteins that changed their abundance upon infection with *R. conorii* or *R. montanensis*. Host proteins that change their abundance in the same direction (increase or decrease abundance) upon infection with both *R. conorii* and *R. montanensis* are considered to be a common response to infection. On the other hand, host proteins that change their abundance in only one infection condition, but show unchanged protein levels in the other, are considered to be a species-specific host response. DOWN(RC), yellow—proteins that are in decreased abundance in *R. conorii*-infected THP-1 macrophages compared to uninfected THP-1 macrophages; UP(RC), blue—proteins that are in increased abundance in *R. conorii*-infected THP-1 macrophages compared to uninfected THP-1 macrophages; DOWN(RM), red—proteins that are in decreased abundance in *R. montanensis*-infected THP-1 macrophages compared to uninfected THP-1 macrophages; UP(RM), green—proteins that are in increased abundance in *R. montanensis*-infected THP-1 macrophages compared to uninfected THP-1 macrophages. Individual information about the proteins in each group of the Venn diagram can be found in Supplementary Table 3. Venn diagrams were obtained using VENNY 2.1 (http://bioinfogp.cnb.csic.es/tools/venny/).

To start distinguishing between common and species-specific host responses to infection, we have sorted these proteins into several groups (Figure 2 and Supplementary Table 3). As illustrated in the Venn diagram, infection of THP-1 macrophages with *R. conorii* or *R. montanensis* resulted in common changes in protein content of 245 host proteins, corresponding to 52 proteins with increased abundance and 193 proteins with reduced abundance shared between infection conditions. Infection with *R. conorii* resulted in specific alterations in the content of 136 host proteins. Of these, 123 proteins were enriched, while 13 showed reduced abundance. On the other hand, *R. montanensis*-specific alterations were observed in 109 proteins, with 11 proteins found in increased abundance and 98 in decreased abundance. We also identified four proteins that are inversely altered in both experimental conditions, with three proteins being overrepresented in *R. conorii*-infected cells and underrepresented in *R. montanensis*-infected THP-1 macrophages, and one protein showing the reverse accumulation pattern.

**Figure 3:**
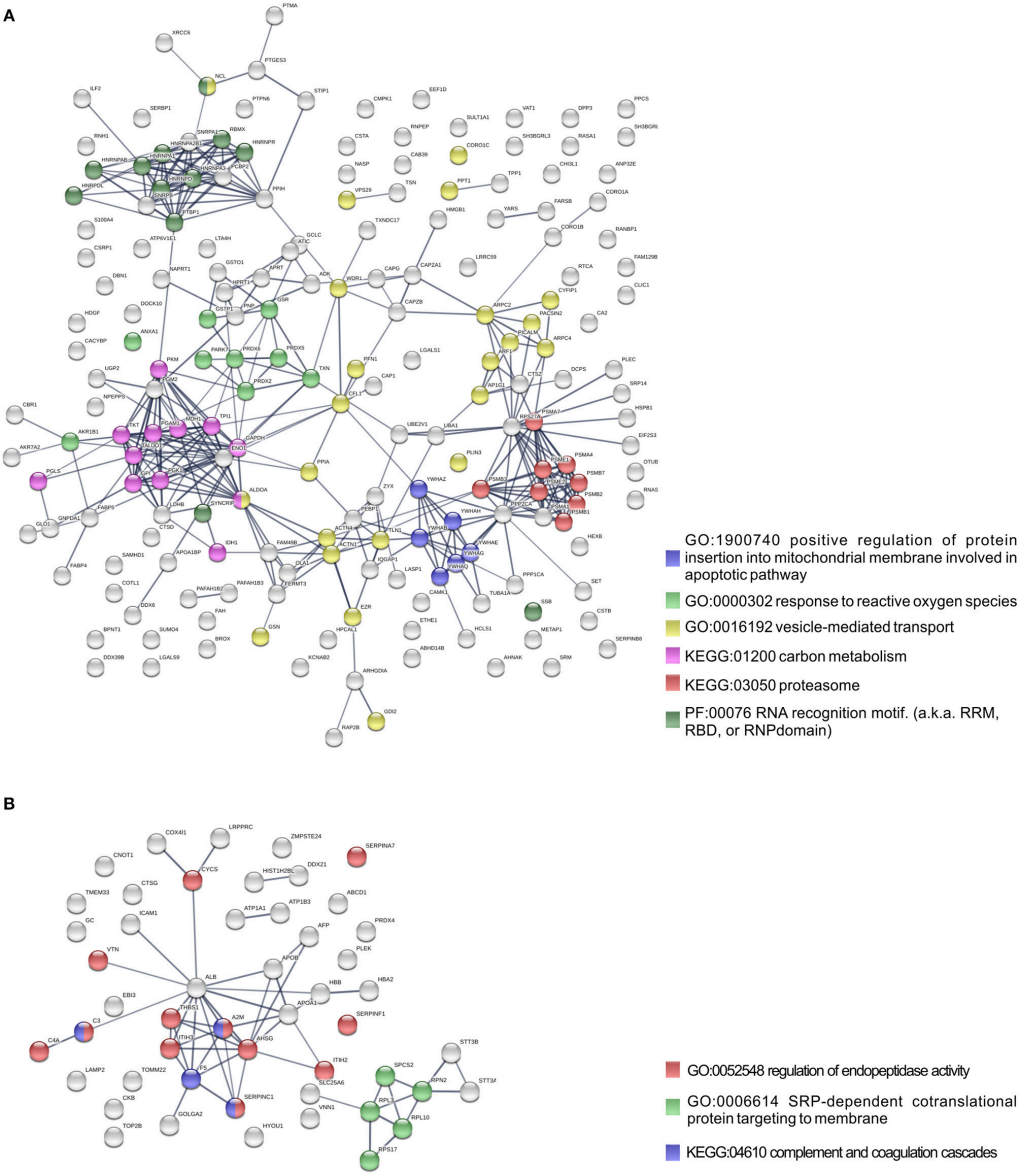
Protein network analysis of common responses to infection with both *R. conorii* and *R. montanensis*. **(A,B)** Clustering of protein-protein interaction networks for the 193 and 52 host proteins commonly altered between infection conditions, found with reduced abundance **(A)** or increased abundance **(B)**, respectively. List of the individual host proteins for each independent analysis can be found in Supplementary Table 3. The analysis was carried out with STRING 10.5 (http://string-db.org/) using high confidence (0.7) score. Nodes are represented with different colors according to their categorization in gene ontology (GO) terms, KEGG pathways or PFAM protein domains.

**Figure 4:**
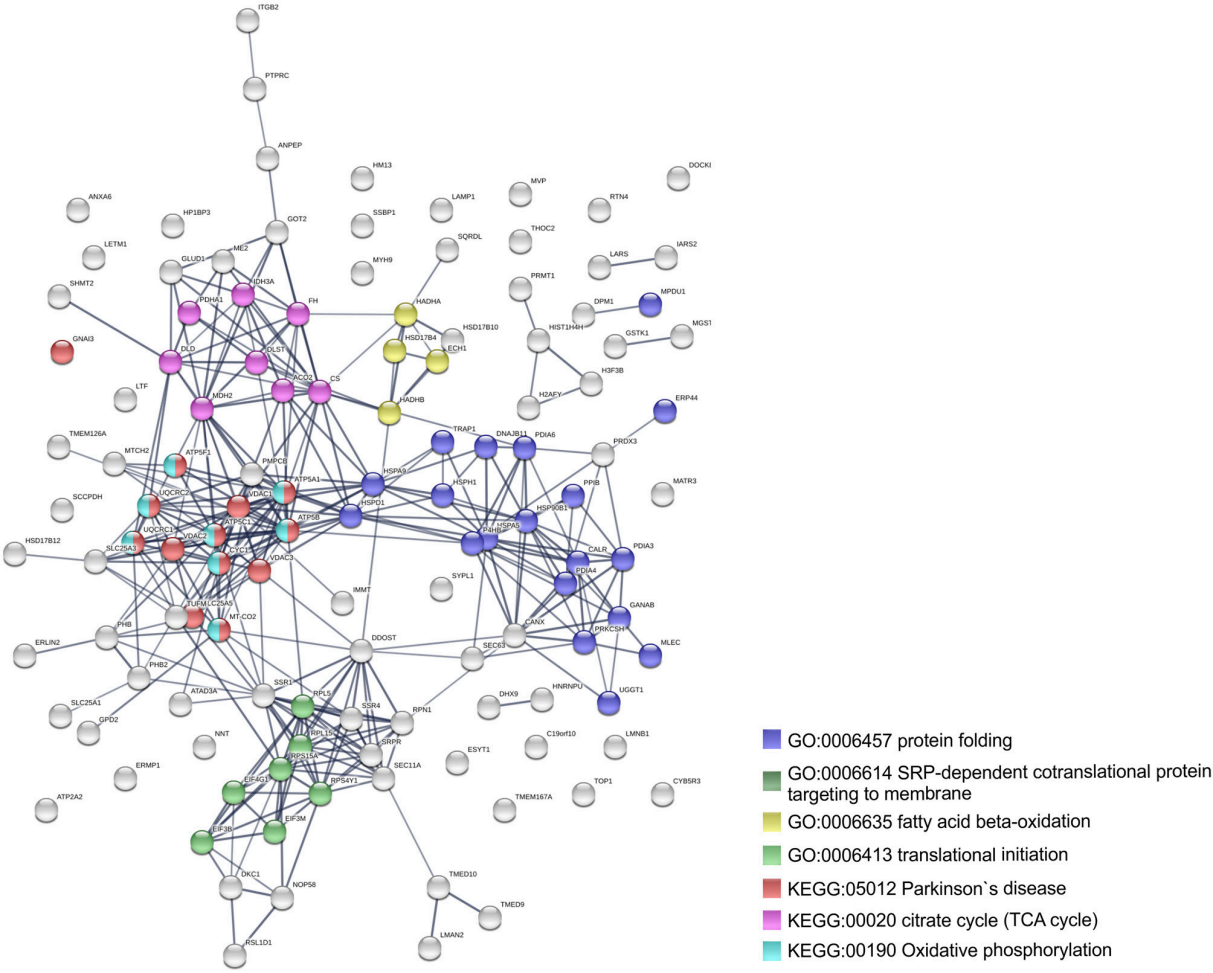
Clustering of host proteins that specifically increase their abundance upon infection with *R. conorii*. Protein-protein interaction network for the 123 host proteins with increased abundance upon infection with *R. conorii*, but unchanged levels upon infection with *R. montanensis*. List of the individual host proteins for each independent analysis can be found in Supplementary Table 3. The analysis was carried out with STRING 10.5 (http://string-db.org/) using high confidence (0.7) score. Nodes are represented with different colors according to their categorization in gene ontology (GO) terms or KEGG pathways.

**Figure 5:**
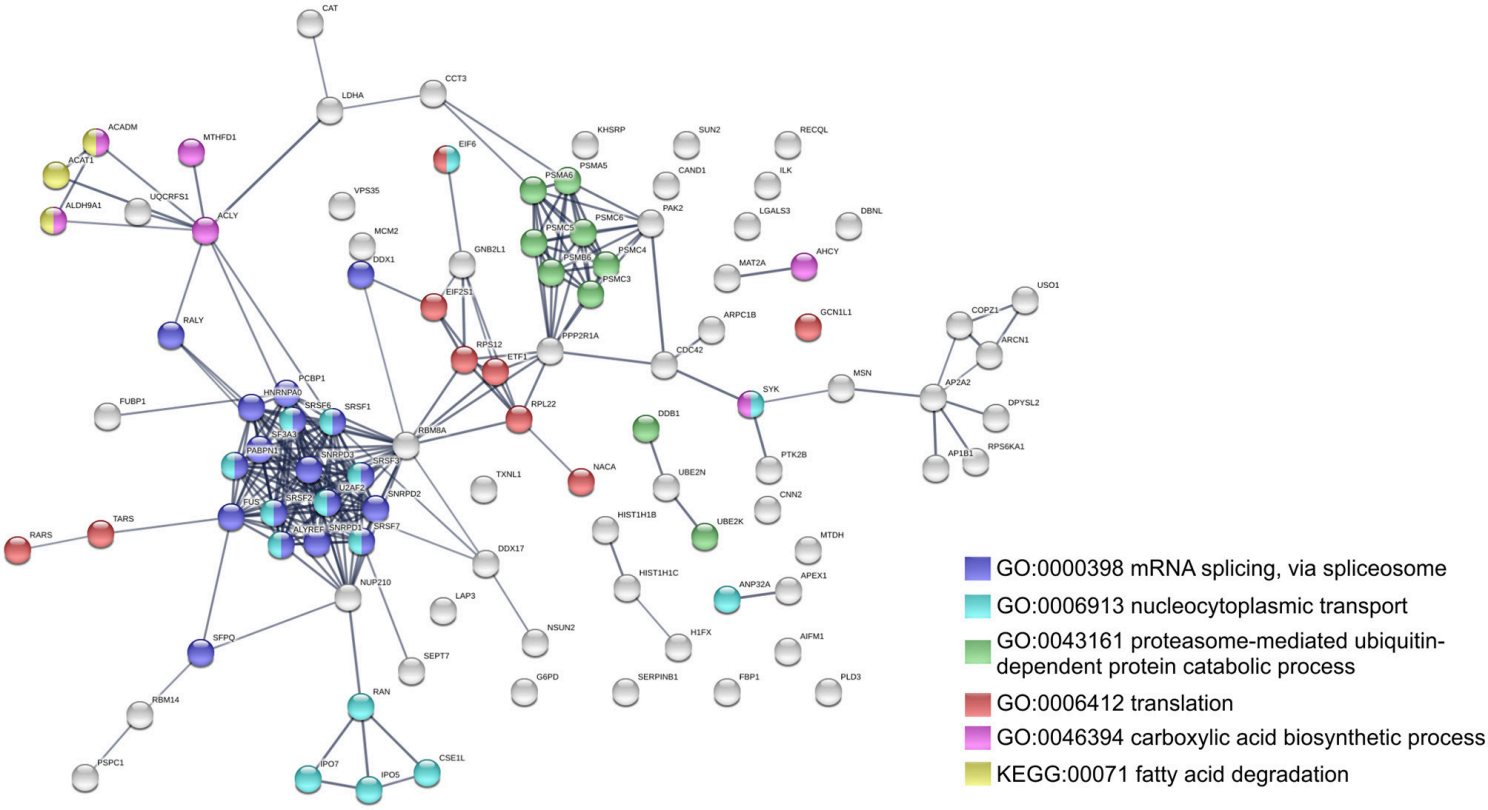
Clustering of host proteins that specifically decrease their abundance upon infection with *R. montanensis*. Protein-protein interaction network for the 98 host proteins with decreased abundance upon infection with *R. montanensis*, but unchanged protein levels upon infection with *R. conorii*. List of the individual host proteins for each independent analysis can be found in Supplementary Table 3. The analysis was carried out with STRING 10.5 (http://string-db.org/) using high confidence (0.7) score. Nodes are represented with different colors according to their categorization in gene ontology (GO) terms or KEGG pathways.

To further identify host processes and molecular pathways that were differentially altered in common and species-specific responses to infection, we performed a Search Tool for Retrieval of Interacting Genes/Proteins (STRING) analysis (Szklarczyk et al., 2017). The global interaction networks obtained for all proteins commonly altered between infection conditions revealed several clusters (Figure 3), which were particularly evident among proteins with reduced abundance (Figure 3A). In this particular functional network, these clusters included GO and KEGG pathway IDs associated with carbon metabolism (KEGG:01200), proteasome (KEGG:03050), positive regulation of protein insertion into mitochondrial membrane involved in apoptotic signaling pathway (GO:1900740), response to reactive oxygen species (GO:0000302), vesicle-mediated transport (GO:0016192), and RNA recognition motif (PF00076), suggesting a common impact of infection with either the pathogenic and non-pathogenic member of SFG *Rickettsia* in different biological processes. For the 52 commonly enriched proteins, the STRING analysis revealed clusters of proteins associated with regulation of endopeptidase activity (GO:0052548), SRP-dependent cotranslational protein targeting to membrane, and complement and coagulation cascades (KEGG:04610) (Figure 3B). Regarding species-specific induced alterations, the obtained interaction networks are shown in Figures 4, 5, and Supplementary Figure 2. The 123 host proteins found in increased abundance in THP-1 macrophages infected with *R. conorii* (Figure 4) clustered in diverse cellular functions, such as protein folding (GO:0006457), SRP-dependent co-translational protein targeting to membrane (GO:0006614), fatty acid beta-oxidation (GO:0006635), translational initiation (GO:0006413), TCA cycle (KEGG:00020), and oxidative phosphorylation (KEGG:00190). These results suggest a significant impact on the modulation of different host metabolic processes specifically induced by *R. conorii*, on top of the cluster for carbon metabolism already observed for shared proteins with reduced abundance (Figure 3A). For the 98 host proteins found specifically in decreased abundance in *R. montanensis*-infected cells (Figure 5), main clusters associated with mRNA splicing via spliceosome (GO:0000398), nucleocytoplasmic transport (GO:0006913), proteasome-mediated ubiquitin-dependent protein catabolic process (GO:0043161), translational (GO:0006412), carboxylic acid biosynthetic process (GO:0046394), and fatty acid degradation (KEGG:00071). For the other two groups of proteins uniquely altered by each rickettsial species, no significant clustering was detected (Supplementary Figure 2).

### A Pathogen and a Non-pathogen SFG *Rickettsia* Trigger Differential Metabolic Signatures in Macrophage-Like Cells

Our proteomics results revealed that different host proteins involved in various metabolic processes were differentially altered upon infection of THP-1 macrophages with either *R. conorii* or *R. montanensis*. Central metabolic pathways such as glycolysis, pentose phosphate pathway (PPP), tricarboxylic acid (TCA) cycle, oxidative phosphorylation (OXPHOS), fatty acid metabolism, and amino acid metabolism were among the processes where significant alterations were observed (Table 3).

**Table 3:** Quantified host proteins involved in several metabolic processes (glycolysis, pentose phosphate pathway, TCA cycle, lipid metabolism, and oxidative phosphorylation) and their respective fold change in abundance upon infection of THP-1 macrophages with *R. conorii* (Rc/uninf) or *R. montanensis* (Rm/uninf).

**Pathway**	**Name**	**UniProt**	**EC number**	**Log_**2**_ (Rc/uninf)**	**Log_**2**_ (Rm/uninf)**
Glycolysis	Phosphoglucomutase 2 (PGM2)	Q96G03	5.4.2.2	−0.59	−0.64
	Glucose-6-phosphate isomerase (GPI)	P06744	5.3.1.9	−0.41	−0.40
	Fructose-1,6-biphosphatase I (FBP1)	P09467	3.1.3.11	−0.04	−0.33
	Phosphofructokinase, liver type (PFKL)	P17858	2.7.1.11	0.09	−0.15
	Aldolase, fructose-biphosphate A (ALDOA)	P04075	4.1.2.13	−0.39	−0.30
	Triosephosphate isomerase (TPI1)	P60174	5.3.1.11	−0.48	−0.40
	Glyceraldehyde-3-phosphate dehydrogenase (GADPH)	P04406	1.2.1.12	−0.56	−0.42
	Phosphoglycerate kinase 1 (PGK1)	P00558	2.7.2.3	−0.60	−0.62
	Phosphoglycerate mutase 1 (PGAM1)	P18669	5.4.2.11	−0.75	−0.60
	Enolase 1 (ENO1)	P06733	4.2.1.11	−0.48	−0.39
	Pyruvate kinase, muscle (PKM)	P14618	2.7.1.40	−0.45	−0.47
	Lactate dehydrogenase A(LDHA)	P00338	1.1.1.27	−0.24	−0.38
	Lactate dehydrogenase B(LDHB)	P07195	1.1.1.27	−0.27	−0.38
Pentose Phosphate Pathway	Glucose-6-phosphate dehydrogenase (G6PD)	P11413	1.1.149/1.1.1.343	−0.22	−0.29
	6-phosphogluconolactonase (PGLS)	O95336	3.1.1.31	−0.80	−0.52
	Phosphogluconate dehydrogenase (PGD)	P52209	1.1.1.44/1.1.1.343	−0.48	−0.25
	Glucose-6-phosphate isomerase (GPI)	P06744	5.3.1.9	−0.41	−0.40
	Transketolase (TKT)	P29401	2.2.1.1	−0.64	−0.60
	Transaldolase 1 (TALDO1)	P37837	2.2.1.2	−0.57	−0.43
	Phosphofructokinase, liver type (PFKL)	P17858	2.7.1.11	0.09	−0.15
	Fructose-biphosphatase 1 (FBP1)	P09467	3.1.3.11	−0.04	−0.33
	Aldolase, fructose-biphosphatase A (ALDOA)	P04075	4.1.2.13	−0.39	−0.30
	Phosphoglucomutase 2 (PGM2)	Q96G03	5.4.2.7/5.4.2.2	−0.59	−0.64
TCA cycle	Pyruvate dehydrogenase (lipoamide) alpha 1 (PDHA1)	P08559	1.2.4.1	0.40	0.17
	ATP citrate lyase (ACLY)	P53396	2.3.3.8	−0.23	−0.37
	Citrate synthase (CS)	O75390	2.3.3.1	0.49	0.14
	Aconitase 2 (ACO2)	Q99798	4.2.1.3	0.36	0.02
	Isocitrate dehydrogenase (NADP(+)) 2, mitochondrial (IDH2)	P48735	1.1.1.42	0.23	−0.04
	Isocitrate dehydrogenase 3 (NAD(+)) apha (IDH3A)	P50213	1.1.1.41	0.42	−0.17
	Isocitrate dehydrogenase (NADP(+)) 1, cytosolic (IDH1)	O75874	1.1.1.42	−0.55	−0.80
	Fumarate hydratase (FH)	P07954	4.2.1.2	0.39	0.11
	Malate dehydrogenase 2 (MDH2)	P40926	1.1.1.37	0.46	0.01
	Malate dehydrogenase 1 (MDH1)	P40925	1.1.1.37	−0.67	−0.62
	Dihydrolipoamide S-acetyltransferase (DLAT)	P10515	2.3.1.12	−0.03	0.17
	Dihydrolipoamide dehydrogenase (DLD)	P09622	1.8.1.4	0.58	−0.11
	Dihydrolipoamide S-succinyltransferase (DLST)	P36957	2.3.1.61	0.44	0.11
	Glutamate dehydrogenase 1 (GLUD1)	P00367	1.4.1.3	0.28	−0.02
	Glutamic-oxaloacetic transaminase 2 (GOT2)	P00505	2.6.1.1	0.48	0.12
	Malic enzyme 2 (ME2)	P23368	1.1.1.38	0.45	0.00
Lipid metabolism	Hydroxyacyl-CoA dehydrogenase/3-ketoacyl-CoA thiolase/enoyl-CoA hydratase (trifunctional protein), beta subunit (HADHB)	P55084	2.3.1.16	0.32	−0.13
	hydroxyacyl-CoA dehydrogenase (HADH)	Q16836	1.1.1.35	0.20	0.13
	Hydroxyacyl-CoA dehydrogenase/3-ketoacyl-CoA thiolase/enoyl-CoA hydratase (trifunctional protein), alpha subunit (HADHA)	P40939	1.1.1.211/4.2.1.17	0.55	0.14
	Palmitoyl-protein thioesterase 1 (PPT1)	P50897	3.1.2.22	−1.34	−0.87
	Hydroxysteroid 17-beta dehydrogenase 12 (HSD17B12)	Q53GQ0	1.1.1.330	0.50	0.25
	Carnitine palmitoyltransferase 2 (CPT2)	P23786	2.3.1.21	0.25	−0.10
	Acyl-CoA dehydrogenase, C-4 to C-12 straight chain (ACADM)	P11310	1.3.8.7	0.12	−0.33
	Acyl-CoA dehydrogenase, very long chain (ACADVL)	P49748	1.3.8.9	0.25	0.07
	Fatty acid synthase (FASN)	P49327	2.3.1.41	0.14	0.08
	Apolipoprotein A1 (APOA1)	P02647	–	1.24	1.35
	Apolipoprotein B (APOB)	P04114	–	1.35	0.54
Electron chain reaction (complex III)	Cytochrome c1 (CYC1)	P08574	1.10.2.2	0.34	0.17
	Ubiquinol-cytochrome c reductase core protein I (UQCRC1)	P31930	1.10.2.2	0.55	0.23
	Ubiquinol-cytochrome c reductase core protein II (UQCRC2)	P22695	1.10.2.2	0.38	0.15
	ubiquinol-cytochrome c reductase, Rieske iron-sulfur polypeptide 1 (UQCRFS11)	P47985	1.10.2.2	−0.16	−0.46
Electron chain reaction (complex IV)	Cytochrome c oxidase subunit 4I1 (COX4I1)	P13073	1.9.3.1	0.42	0.85
	Cytochrome c oxidase subunit II (COX2)	P00403	1.9.3.1	0.36	0.12
Electron chain reaction (complex V)	ATP synthase, H+ transporting, mitochondrial F1 complex, alpha subunit 1, cardiac muscle (ATP5A1)	P25705	3.6.3.14	0.43	−0.05
	ATP synthase, H+ transporting, mitochondrial F1 complex, beta polypeptide (ATP5B)	P06576	3.6.3.14	0.47	0.10
	ATP synthase, H+ transporting, mitochondrial F1 complex, gamma polypeptide 1 (ATP5C1)	P36542	3.6.3.14	0.39	−0.05
	ATP synthase, H+ transporting, mitochondrial F1 complex, delta subunit (ATP5D)	P30049	3.6.3.14	0.20	0.12
	ATP synthase, H+ transporting, mitochondrial Fo complex subunit B1 (ATP5F1)	P24539	3.6.3.14	0.35	0.24
	ATPase H+ transporting V1 subunit A (ATP6V1A)	P38606	3.6.3.14	0.08	0.06
	ATPase H+ transporting V1 subunit E1 (ATP6V1E1)	P36543	3.6.3.14	−0.33	−0.36
	ATP synthase, H+ transporting, mitochondrial F1 complex, O subunit (ATP5O)	P48047	3.6.3.14	0.09	0.31
	ATPase H+ transporting V0 subunit d1 (ATP6V0D1)	P61421	3.6.3.14	0.15	−0.11
	ATP synthase, H+ transporting, mitochondrial Fo complex subunit F2 (ATP5J2)	P56134	3.6.3.14	0.10	−0.11

*Proteins that are considered as altered (fold change ≤ 0.83 or fold change ≥ 1.2) between infected and uninfected conditions were color-coded according the following: decreased (blue), not changed (transparent), or increased (orange)*.

**Figure 6:**
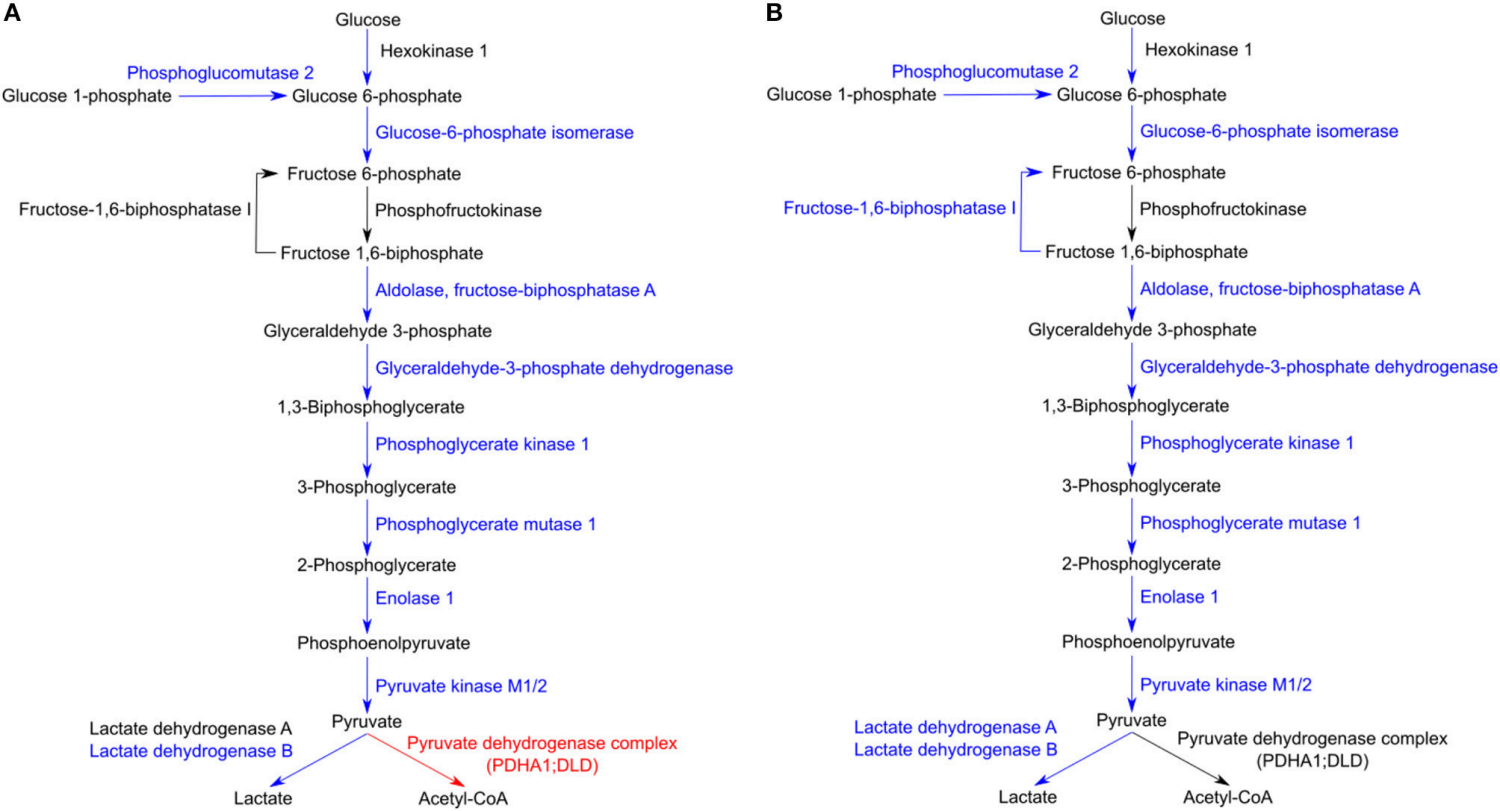
Host glycolytic enzymes found in reduced abundance upon infection of THP-1 macrophages with both rickettsial species. **(A,B)** Infection of THP-1 macrophages with either *R. conorii* **(A)** or *R. montanensis* **(B)** resulted in a decrease in the abundance of several host glycolytic enzymes at 24 h post-infection. UniProt accession number and the respective fold change upon infection can be found in Table 3 for each respective enzyme. Enzymes with unchanged or decreased protein levels, when compared to uninfected cells, are represented in black and blue, respectively. Red represents enzymes of the pyruvate dehydrogenase complex found accumulated in *R. conorii*-infected cells (Table 3).

Regarding glycolysis, the majority of the enzymes involved in the different steps of glucose conversion to pyruvate (names and reactions catalyzed shown in Figures 6A,B), and lactate dehydrogenase B (LDHB; P07195) which catalyzes the interconversion of pyruvate and lactate in a post-glycolytic process, were found significantly reduced in abundance under both infection conditions. A similar pattern was observed for several enzymes involved in the PPP (Table 3). As the glycolytic metabolism, this pathway assumes a key role by providing intermediates that serve other critical anabolic and catabolic processes (Stincone et al., 2015), with our results suggesting a reduced activity of both metabolic pathways in response to rickettsial infection.

**Figure 7:**
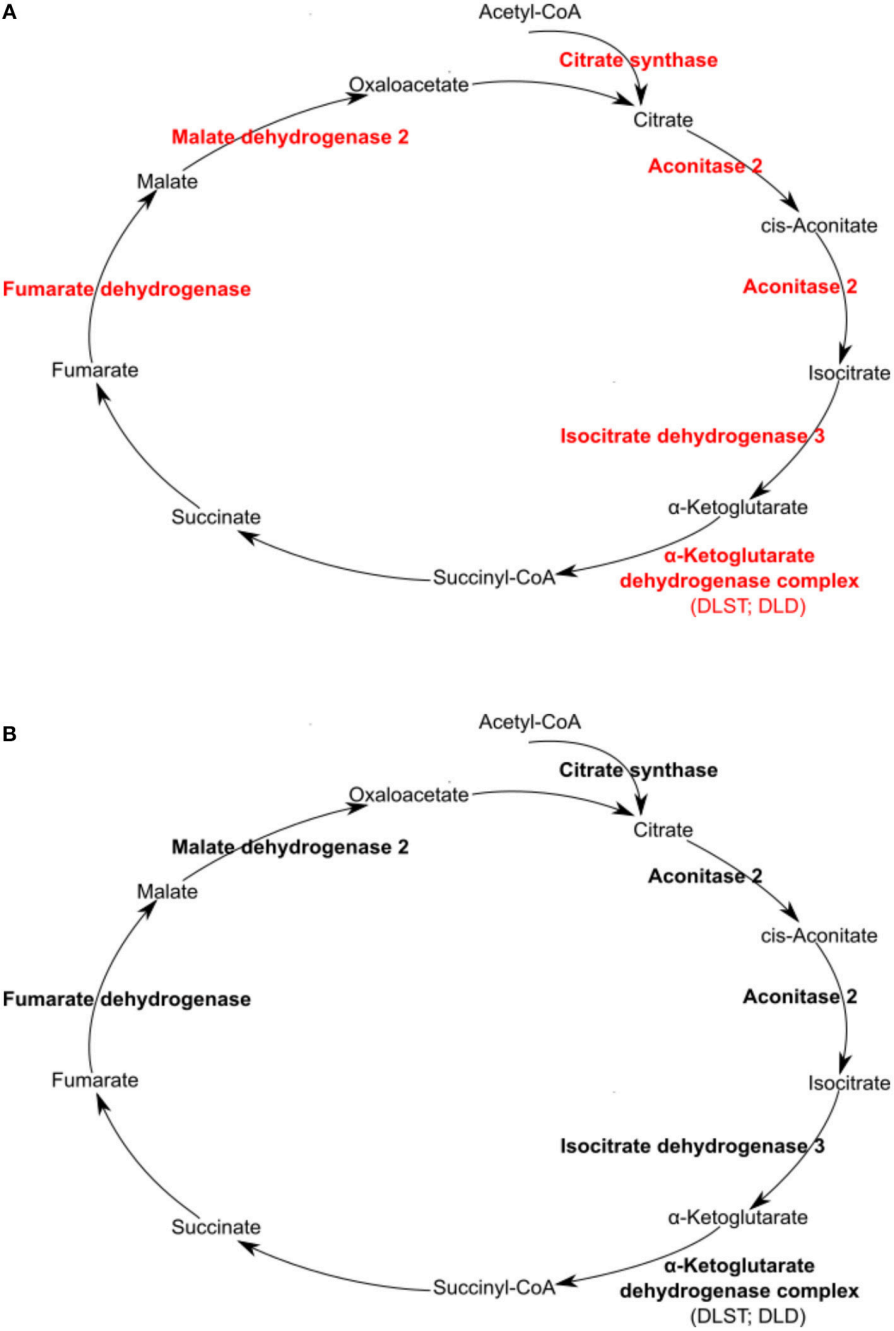
*Rickettsia conorii*, but not *R. montanensis*, infection increased the abundance of several enzymes of the TCA cycle in THP-1 macrophages. Infection of THP-1 macrophages with either *R. conorii* **(A)** or *R. montanensis* **(B)** resulted in alterations in the abundance of several host TCA cycle enzymes at 24 h post-infection. UniProt accession number and the respective fold change upon infection can be found in Table 3 for each respective enzyme. Enzymes with unchanged or increased protein levels, when compared to uninfected cells, are represented in black and red, respectively.

A differential macrophage response to *R. conorii*- vs. *R. montanensis*-challenge was observed for proteins implicated in other key metabolic pathways (Table 3). Several TCA cycle enzymes were found enriched only in THP-1 macrophages infected with *R. conorii* (Figures 7A,B). More specifically, infection with *R. conorii* resulted in the increased abundance of citrate synthase (CS, O75390), the enzyme that catalyzes the condensation of acetyl-CoA and oxaloacetate to form citrate; aconitase (ACO2, Q99798), that catalyzes the isomerization of citrate to isocitrate; isocitrate dehydrogenase 3 (IDH3A, P50213), the enzyme responsible for the oxidative decarboxylation of isocitrate to α-ketoglutarate; fumarate hydratase (FH, P07954), which catalyzes the reversible hydration/dehydration of fumarate to malate; and malate dehydrogenase 2 (MDH2, P40926) that catalyzes the reversible oxidation of malate to oxaloacetate (Figure 7A). Two additional enzymes were found in reduced abundance under both infection conditions, isocitrate dehydrogenase 1 (IDH1, O75874) that catalyzes the oxidative decarboxylation of isocitrate to α-ketoglutarate (in the cytoplasm), and malate dehydrogenase 1 (MDH1, P40925) that catalyzes the reversible cytoplasmic conversion of oxaloacetate to malate. ATP citrate lyase (ACLY, P53396), which catalyzes the cytosolic formation of acetyl-CoA and oxaloacetate from citrate and CoA, was found in significantly reduced abundance only in *R. montanensis*-infected cells (Table 3).

The TCA cycle coupled to OXPHOS constitute a highly efficient mode for ATP generation, providing for basal subsistence in most cells types (O'Neill et al., 2016). We found that infection of THP-1 macrophages with *R. conorii* resulted in the increased abundance of several proteins of the complex III, IV, and V of the electron transport chain. More specifically, *R. conorii*-infected cells revealed an increased abundance of cytochrome C1 (CYC1, P08574), ubiquinol-cytochrome c reductase core protein 1 (UQCRC1, P31930), and ubiquinol-cytochrome c reductase core protein 2 (UQCRC2, P22695), that are members of the complex III. Members of complex IV, such as cytochrome c oxidase subunit 4l1 (COX4l1, P13073) and cytochrome c oxidase subunit II (COX2, P00403), together with several subunits of F-type ATPase (complex V) also accumulated in *R. conorii*-infected cells (Table 3). The observed increase in abundance of several TCA cycle and OXPHOS enzymes in *R. conorii*-infected macrophages suggests differences in the metabolic requirements of infected cells.

Although host glycolytic enzymes were found in reduced abundance upon infection with *R. conorii* and *R. montanensis*, we observed significant differences between datasets in the content of proteins involved in mitochondrial pyruvate conversion as well as in lipid and glutamate metabolism (Table 3). These results suggest that alternative carbon sources may be involved in TCA cycle feeding and that these may be differentially modulated by each bacterial species. Among *R. conorii*-specific responses, we found increased abundance of malic enzyme 2 (ME2, P23368), which catalyzes the oxidative decarboxylation of mitochondrial malate to pyruvate, and of proteins of the pyruvate dehydrogenase complex which irreversibly converts pyruvate to acetyl-CoA [pyruvate dehydrogenase E1 alpha 1 subunit (PDHA1, P08559) and dihydrolipoamide dehydrogenase (DLD, P09622)] (Figure 6), suggesting the formation of mitochondrial pyruvate that may re-enter the TCA cycle by conversion to acetyl-CoA.

Moreover, several enzymes involved in fatty acid oxidation were differentially altered between infection conditions. Subunits alpha and beta of the mitochondrial trifunctional protein (HADHA, P40939; HADHB, P55084) that catalyze three out of four steps in mitochondrial β-oxidation, and Δ(3,5)- Δ(2,4)-dienoyl-CoA isomerase (ECH1, Q13011) that functions in the auxiliary step of β-oxidation are enriched only in *R. conorii*-infected THP-1 macrophages (Table 3). The bifunctional enzyme HSD17B4, known as peroxisomal multifunctional enzyme type 2 (P51659) that acts on the peroxisomal β-oxidation, also specifically accumulated in *R. conorii*-infected cells (Table 3), suggesting an increase of activity of both mitochondrial and peroxisomal β-oxidation. Overall, the accumulation of fatty acid β-oxidation enzymes in *R. conorii*-infected cells may indicate an increase of β-oxidation activity to generate acetyl-CoA from lipids, which can then be used to feed the TCA cycle to increase ATP production via OXPHOS. As noted previously, several TCA cycle and OXPHOS enzymes are indeed found in increased abundance in this dataset only. The lipid transport proteins apolipoprotein A1 (APOA1, P02647) and apolipoprotein B (APOB, P04114) were overrepresented in both *R. conorii*- and *R. montanensis*-infected cells. Apolipoproteins are reported to influence inflammatory responses, with APOA1 known to display anti-inflammatory functions (Burger and Dayer, 2002; Sirnio et al., 2017).

**Figure 8:**
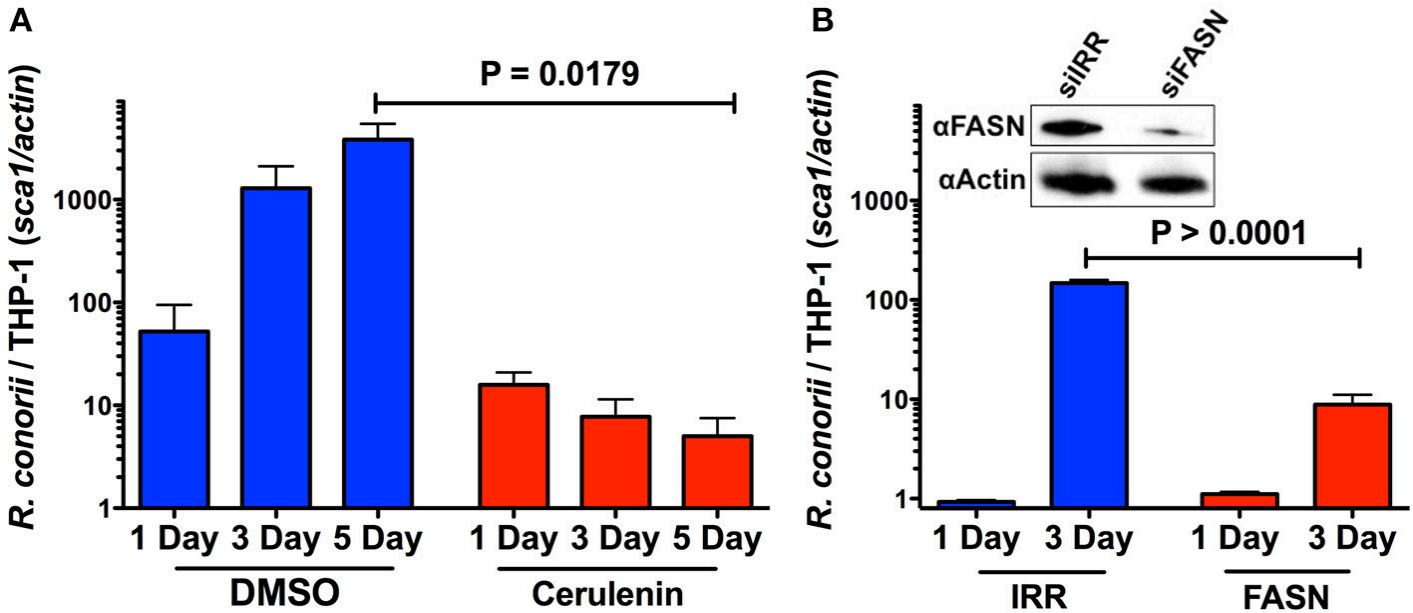
Inhibition of FASN function and expression inhibits the ability of *R. conorii* to proliferate within THP-1 cells. **(A)** Quantitative PCR analysis of gDNA from PMA differentiated THP-1 cells infected with *R. conorii* Malish7 in the presence of DMSO (vehicle control) or FASN inhibitor, cerulenin, at 24 h, 3, and 5 days-post treatment. (**B**, Inset) Western immunoblot analysis confirms the inhibition of FASN protein expression from samples isolated at 3 days-post transfection when compared to control RNA treated cells (siIRR). An immunoblot for Actin is used to control for equal protein loading in each lane. Quantitative PCR analysis of gDNA from PMA differentiated THP-1 cells transfected with siRNA against FASN or a control RNA and infected with *R. conorii* Malish7 at an MOI of 2. Samples were analyzed at 24 h and 3 days post infection. Data is representative of two independent experiments with each condition in triplicate. Statistical analysis was performed by One-Way ANOVA with a Dunnett's *post-hoc* test for pairwise comparison. Statistical significance (*p* < 0.05).

We have demonstrated that infection of THP-1 cells with *R. conorii* leads to an increase in the overall expression of proteins that are involved in host lipid metabolism. Therefore, we hypothesized that *R. conorii* may also require *de novo* fatty acid synthesis for successful proliferation and survival in macrophage-like cells. To test this, we first selected an upstream enzyme, FASN, which is involved in the anabolism of fatty acids necessary for the function of a wide range of lipid metabolic processes. The level of *R. conorii* growth was quantified by qPCR at 24 h, 3, and 5 days in the presence of a pharmacological inhibitor of FASN, cerulenin. As shown in Figure 8A, the ability of *R. conorii* to proliferate in the presence of cerulenin when compared to the DMSO control was significantly reduced at 5 days post-infection.

To ensure the inhibition of *R. conorii* proliferation seen in the presence of cerulenin was due to the inhibition of FASN, we sought to silence the expression of FASN in THP-1 cells and then determine subsequent *R. conorii* growth within these cells. We transfected cells using a specific siRNA targeting *FASN* and compared the expression of fatty acid synthase in cells transfected with an irrelevant control siRNA. As shown in Figure 8B, (inset), transfection of *FASN* siRNA is effective in reducing total fatty acid synthase expression in differentiated THP-1 cells. The growth of *R. conorii* in si*FASN*-transfected differentiated THP-1 cells is significantly inhibited at 3 days post-infection when compared to growth in control transfected cells (Figure 8B). Together, these results indicate that the activity and expression of host fatty acid synthase are required for successful growth of *R. conorii* in macrophage-like cells.

The TCA cycle can also use glutamate as an important anaplerotic substrate. Strikingly, glutamate dehydrogenase 1 (GLUD1, P00367) that converts glutamate into α-ketoglutarate and mitochondrial aspartate transaminase (GOT2, P00505), which generates α-ketoglutarate and aspartate from glutamate and oxaloacetate, are both in increased abundance in *R. conorii*-infected cells only. Moreover, proteins of the α-ketoglutarate dehydrogenase complex [dihydrolipoamide S-succinyltransferase (DLST, P36957) and dihydrolipoamide dehydrogenase (DLD, P09622)], which catalyzes the conversion of α-ketoglutarate to succinyl-CoA, were also enriched specifically in this dataset. Globally, *R. conorii* appears to positively interfere with different pathways that generate intermediates to feed the TCA cycle (mitochondrial pyruvate conversion, fatty acid oxidation, and glutamate metabolism).

Moreover, proteins found specifically in increased abundance in *R. conorii*-infected cells include different types of inner and outer membrane localized transporters (Table 4). Namely, four members of solute carrier family 25 (SLC25): SCL25A1 (P53007), the citrate (tricarboxylate) carrier which transports citrate out of the mitochondria (exchange with malate); SLC25A3 (Q00325), which transports phosphate groups from the cytosol to the mitochondrial matrix (cotransport protons), and SCL25A5 (P05141) and SLC25A6 (P12236) that catalyze the exchange of ADP and ATP across the mitochondrial inner membrane; mitochondrial carrier 2 (MTCH2, Q9Y6C9) (whose transported substrate is still unknown); the outer membrane voltage-dependent anion channels VDAC1 (P21796), VDAC2 (P45880), VDAC3 (Q9Y277), which permeate different small hydrophilic molecules; and two components of the preprotein translocase complex of the outer mitochondrial membrane (TOM complex) [(TOMM22, Q9NS69) and (TOMM40, O96008)]. This accumulation of different mitochondrial transporters is again suggestive of changes in metabolic supply and demand, which appear to be specifically induced by the pathogenic *R. conorii*.

**Table 4:** Quantified host proteins categorized as mitochondrial transporters and their respective fold change in abundance upon infection of THP-1 macrophages with *R. conorii* (Rc/uninf) or *R. montanensis* (Rm/uninf).

**Pathway**	**Name**	**UniProt**	**Log_**2**_ (Rc/uninf)**	**Log_**2**_ (Rm/uninf)**
**Mitochondrial transporters**	Voltage dependent anion channel 1 (VDAC1)	P21796	0.49	−0.01
	Voltage dependent anion channel 2 (VDAC2)	P45880	0.37	0.02
	Voltage dependent anion channel 3 (VDAC3)	Q9Y277	0.42	0.10
	Solute carrier family 25 member 1 (SLC25A1)	P53007	0.38	0.07
	Solute carrier family 25 member 3 (SLC25A3)	Q00325	0.66	0.14
	Solute carrier family 25 member 5 (SLC25A5)	P05141	0.52	0.24
	Solute carrier family 25 member 6 (SLC25A6)	P12236	0.62	0.36
	Solute carrier family 25 member 11 (SLC25A11)	Q02978	0.11	0.22
	Translocase of outer mitochondrial membrane 22 (TOMM22)	Q9NS69	0.55	0.29
	Translocase of outer mitochondrial membrane 40 (TOMM40)	O96008	0.32	−0.29
	Mitochondrial carrier 2 (MTCH2)	Q9Y6C9	0.41	0.14

*Proteins that are considered as altered (fold change ≤ 0.83 or fold change ≥ 1.2) between infected and uninfected conditions were color-coded according the following: decreased (blue), not changed (transparent) or increased (orange)*.

Several proteins involved in different metabolic processes were chosen for follow-up validation by Western blot analysis (Supplementary Figure 3), with results showing the same trend in alterations in protein abundance between the quantitative data from SWATH-MS and the Western blot results. Moreover, Western blot analysis of one protein from complex I (NDUFB8) and one from complex II (SDHB) of the electron transport chain, that were not quantified in our dataset, revealed an accumulation of both proteins in *R. conorii*-infected cells (Supplementary Figure 3). These results are consistent with the *R. conorii*-specific augmented accumulation of OXPHOS proteins from complexes III, IV, and V observed in the quantitative SWATH-MS data.

### Differential Reprogramming of Host Protein Processing Machinery by SFG *Rickettsia* Species

The proteasome is the central proteolytic complex of one of the main protein clearance mechanisms that ensures proteostasis in eukaryotic cells [the ubiquitin-proteasome system (UPS)] (Vilchez et al., 2014; Bentea et al., 2017). We herein found that infection of THP-1 macrophages with either *R. conorii* or *R. montanensis* resulted in significant alterations in the protein content of multiple subunits of the proteasome, which were found in reduced abundance when compared to uninfected cells (Table 5). More specifically, from the core particle (20S) of the proteasome, out of the seven α-subunits that are involved in the formation of the two outer rings three were found in lower abundance in the *R. conorii* dataset [subunits α1 (P25786), α4 (P25789), and α7 (O14818)] and five in the *R. montanensis* dataset [subunits α1 (P25786), α4 (P25789), α5 (P28066), α6 (P60900), and α7 (O14818)]. From the outer ring (subunits β1-β7), we observed a decreased abundance of subunits β1 (P20618), β2 (P49721), β3 (P49720), and β7 (Q99436) in both *R. conorii* and *R. montanensis*-infected cells, with the subunit β6 (P28072) found additionally reduced in the latter. Also, *R. montanensis* infection resulted in a specific underrepresentation of four [PSMC3 (P17980), PSMC4 (P43686), PSMC5 (P62195), and PSMC6 (P62333)] out of the six AAA-ATPases that are part of the base of the 19S regulatory particle.

**Table 5:** Quantified host proteins categorized in proteasome and protein processing in endoplasmic reticulum and their respective fold change in abundance upon infection of THP-1 macrophages with *R. conorii* (Rc/uninf) or *R. montanensis* (Rm/uninf).

**Pathway**	**Name**	**UniProt**	**EC number**	**Log_**2**_ (Rc/uninf)**	**Log_**2**_ (Rm/uninf)**
Proteasome	26S proteasome non-ATPase regulatory subunit 1 (PSMD1)	Q99460	–	0.04	−0.01
	26S proteasome non-ATPase regulatory subunit 2 (PSMD2)	Q13200	–	−0.12	−0.25
	26S proteasome non-ATPase regulatory subunit 3 (PSMD3)	O43242	–	0.01	0.02
	26S proteasome non-ATPase regulatory subunit 6 (PSMD6)	Q15008	–	0.07	0.06
	26S proteasome non-ATPase regulatory subunit 11 (PSMD11)	O00231	–	0.11	0.02
	26S proteasome non-ATPase regulatory subunit 12 (PSMD12)	O00232	–	0.22	0.15
	26S proteasome non-ATPase regulatory subunit 13 (PSMD13)	Q9UNM6	–	0.06	−0.21
	26S proteasome non-ATPase regulatory subunit 14 (PSMD14)	O00487	–	−0.11	−0.07
	26S proteasome regulatory subunit 4 (PSMC1)	P62191	–	0.02	−0.04
	26S proteasome regulatory subunit 7 (PSMC2)	P35998	–	−0.10	−0.23
	26S proteasome regulatory subunit 6A (PSMC3)	P17980	–	−0.25	−0.42
	26S proteasome regulatory subunit 6B (PSMC4)	P43686	–	−0.19	−0.45
	26S proteasome regulatory subunit 8 (PSMC5)	P62195	–	−0.06	−0.38
	26S proteasome regulatory subunit 10B (PSMC6)	P62333	–	−0.13	−0.30
	Proteasome subunit alpha type-1 (PSMA1)	P25786	3.4.25.1	−0.32	−0.38
	Proteasome subunit alpha type-2 (PSMA2)	P25787	3.4.25.1	−0.11	−0.09
	Proteasome subunit alpha type-3 (PSMA3)	P25788	3.4.25.1	−0.23	−0.26
	Proteasome subunit alpha type-4 (PSMA4)	P25789	3.4.25.1	−0.41	−0.52
	Proteasome subunit alpha type-5 (PSMA5)	P28066	3.4.25.1	−0.15	−0.34
	Proteasome subunit alpha type-6 (PSMA6)	P60900	3.4.25.1	−0.23	−0.29
	Proteasome subunit alpha type-7 (PSMA7)	O14818	3.4.25.1	−0.36	−0.33
	Proteasome subunit beta type-1 (PSMB1)	P20618	3.4.25.1	−0.37	−0.35
	Proteasome subunit beta type-2 (PSMB2)	P49721	3.4.25.1	−0.34	−0.34
	Proteasome subunit beta type-3 (PSMB3)	P49720	3.4.25.1	−0.35	−0.55
	Proteasome subunit beta type-6 (PSMB6)	P28072	3.4.25.1	−0.24	−0.42
	Proteasome subunit beta type-7 (PSMB7)	Q99436	3.4.25.1	−0.49	−0.43
	Proteasome subunit beta type-8 (PSMB8)	P28062	3.4.25.1	−0.01	−0.10
	Proteasome activator complex subunit 1 (PSME1)	Q06323	–	−0.42	−0.47
	Proteasome activator complex subunit 2 (PSME2)	Q9UL46	–	−0.55	−0.29
Protein processing in endoplasmic reticulum	Signal sequence receptor subunit 1 (SSR1)	P43307	–	0.41	−0.05
	Signal sequence receptor subunit 4 (SSR4)	P51571	–	0.49	0.19
	Dolichyl-diphosphooligosaccharide–protein glycosyltransferase non-catalytic subunit (DDOST)	P39656	–	0.44	0.03
	STT3A, catalytic subunit of the oligosaccharyltransferase complex (STT3A)	P46977	2.4.99.18	0.65	0.30
	STT3B, catalytic subunit of the oligosaccharyltransferase complex (STT3B)	Q8TCJ2	2.4.99.18	0.72	0.34
	Ribophorin I (RPN1)	P04843	–	0.45	0.04
	Ribophorin II (RPN2)	P04844	–	0.51	0.35
	Heat shock protein family A (Hsp70) member 5 (HSPA5)	P11021	–	0.57	−0.02
	Calnexin (CANX)	P27824	–	0.70	0.14
	Calreticulin (CALR)	P27797	–	0.47	0.00
	Hypoxia up-regulated 1 (HYOU1)	Q9Y4L1	–	0.64	0.29
	DnaJ heat shock protein family (Hsp40) member B11 (DNAJB11)	Q9UBS4	–	0.38	−0.19
	Heat shock protein family A (Hsp70) member 8 (HSPA8)	P11142	–	−0.21	−0.12
	Heat shock protein 90 alpha family class A member 1 (HSP90AA1)	P07900	–	−0.21	−0.26
	Heat shock protein 90 beta family member 1 (HSP90B1)	P14625	–	0.56	0.00
	Heat shock protein 90 alpha family class B member 1 (HSP90AB1)	P08238	–	0.14	0.09
	Heat shock protein family H (Hsp110) member 1 (HSPH1)	Q92598	–	0.34	−0.04
	Protein disulfide isomerase family A member 3 (PDIA3)	P30101	5.3.4.1	0.32	0.03
	Protein disulfide isomerase family A member 4 (PDIA4)	P13667	5.3.4.1	0.46	0.04
	Protein disulfide isomerase family A member 6 (PDIA6)	Q15084	5.3.4.1	0.49	0.00
	UDP-glucose glycoprotein glucosyltransferase 1 (UGGT1)	Q9NYU2	2.4.1.	0.43	0.03
	Glucosidase II alpha subunit (GANAB)	Q14697	3.2.1.84	0.43	0.14
	Protein kinase C substrate 80K-H (PRKCSH)	P14314	–	0.38	−0.07
	Lectin, mannose binding 1 (LMAN1)	P49257	–	0.26	0.10
	Lectin, mannose binding 2 (LMAN2)	Q12907	–	0.33	0.01
	SEC13 homolog, nuclear pore and COPII coat complex component (SEC13)	P55735	–	0.08	−0.06
	SEC63 homolog, protein translocation regulator (SEC63)	Q9UGP8	–	0.60	0.19
	Valosin containing protein (VCP)	P55072	–	−0.06	−0.13
	Eukaryotic translation initiation factor 2 subunit alpha (EIF2S1)	P05198	–	−0.17	−0.35

*Proteins that are considered as altered (fold change ≤ 0.83 or fold change ≥ 1.2) between infected and uninfected conditions were color-coded according the following: decreased (blue), not changed (transparent) or increased (orange)*.

A modified type of proteasome called the immunoproteasome is responsible for generating antigen peptides with substantial binding affinity for the major histocompatibility complex I (MHC I) (Kaur and Batra, 2016). The immunoproteasome contains an alternate regulator, known as the PA28 (or 11S), which replaces the 19S regulatory particle and can also activate the core particle. Remarkably, both PA28α (Q06323) and PA28β (Q9UL46)—the α and β immune subunits of the activator PA28—were found in reduced abundance in THP-1 macrophages infected with *R. conorii* and *R. montanensis*. Overall, these results show a significant interference of both rickettsial species with a key regulatory proteolytic machinery, with potential impact in several cellular processes through impaired proteasome activity.

Any condition that decreases proteasome function may result in the accumulation of misfolded proteins within the endoplasmic reticulum (ER), leading to a state known as “ER stress” (Hetz and Papa, 2018). Given the importance of ER-quality control and ER-associated degradation processes for maintaining cellular homeostasis, the cell responds to ER stress with the activation of elaborate compensatory signals to restore ER homeostasis and to ensure cell survival, a process collectively known as ER stress response or unfolded protein response (UPR) (Martins et al., 2016). In this work, we found significant differences in several proteins clustering with protein processing and quality control in ER between infection conditions (Table 5). More specifically, *R. conorii* infection led to the increased abundance of (i) proteins associated with translocation across ER membrane [signal sequence receptor subunits 1 (SSR1, P43307) and 4 (SSR4, P51571)]; (ii) of several subunits of the N-oligosaccharyl transferase (OST) complex [dolichyl-diphosphooligosaccharide-protein glycosyltransferase non-catalytic subunit (DDOST, P39656), OST catalytic subunits STT3A (P46977) and STT3B (Q8TCJ2), ribophorin I (P04843), and ribophorin II (P04844)]; (iii) as well as of several proteins comprising chaperone activity, such as BiP (P11021), calnexin (P27824), calreticulin (P27797), hypoxia up-regulated 1 (Q9Y3L1), endoplasmin (P14625), and DnaJ Heat Shock Protein Family (Hsp40) Member B11 (Q9UBS4). In addition, several protein disulfide isomerases (PDI), such as PDI family A members 3, 4, and 6 (P30101, P13667, Q15084, respectively) were also found in higher abundance in *R. conorii*-infected cells. The same increased abundance pattern was observed for the protein folding sensor UDP-glucose:glycoprotein glucosyltransferase 1 (Q9NYU2), that recognizes glycoproteins with minor folding defects and re-glucosylates them, and glucosidase II alpha subunit (Q14697), another glycan modification enzyme implicated in protein quality control in the ER. In *R. montanensis*-infected cells, with the exception of STT3A (P46977), STT3B (Q8TCJ2), ribophorin II (P04844), HYOU1 (Q9Y4L1), and the eukaryotic translation initiation factor 2 subunit alpha (EIF2S1, P05198) (the latter found in reduced abundance), no significant alterations in abundance were found in the other quantified proteins.

Therefore, this differential accumulation of various proteins involved in protein folding and quality control in the ER anticipates significant differences between bacterial species in their ability to counterbalance ER stress.

## Discussion

A growing body of knowledge has been emerging showing that macrophages can display high plasticity, being able to adopt various activation states (with different metabolic requirements) to accommodate their diverse functional repertoire (Van den Bossche et al., 2017). This metabolic plasticity plays a key role not only in the initiation of the host-cell defense programs aimed to eliminate the invading pathogen, part of a global immune response termed “immunometabolism,” but also in the complex metabolic adaptation reactions that need to take place in both interacting partners for the successful intracellular replication of pathogens, a concept recently coined as “pathometabolism” (Eisenreich et al., 2015, 2017; O'Neill and Pearce, 2016; Van den Bossche et al., 2017). More in-depth knowledge about these mutual metabolic adaptations is growing for different intracellular bacteria (Xavier et al., 2013; Eisenreich et al., 2017) which are known to employ different strategies to modulate host cell metabolism to create a more permissive replication niche (Abu Kwaik and Bumann, 2015; Eisenreich et al., 2017). However, the current state of knowledge on these processes for rickettsial species is rather poor. In this work, we provide evidence that two rickettsial species with different pathogenicity attributes induce differential changes in proteins associated with key metabolic pathways in these cells. Although both *R. conorii* and *R. montanensis*-infection resulted in a lower accumulation of several enzymes of glycolysis and PPP in THP-1 macrophages, the differences in abundance observed in enzymes involved in TCA cycle, OXPHOS, fatty acid oxidation, glutamate metabolism as well as in different mitochondrial transporters, suggest a significant metabolic reprogramming of macrophages specifically induced by the pathogenic *R. conorii*.

Changes in metabolic pathways are known to regulate macrophage activation states and functions. The best studied are likely the two polarized M1 (pro-inflammatory bactericidal) and M2 (anti-inflammatory) subtypes, characterized by different metabolic signatures (Eisenreich et al., 2017). Two metabolic hallmarks of inflammatory M1-like macrophages are increased glycolysis and elevated activity of the PPP. The induction of glycolysis supports pro-inflammatory functions in different ways, including the production of ATP to sustain phagocytic functions as well as feeding of the PPP for nucleotide and protein synthesis, and generation of ROS by NADPH oxidase. Interestingly, our results suggest a decrease in the activity of host glycolysis and reduction in PPP in response to both *R. conorii* and *R. montanensis* at this stage of infection (24 hpi).

Moreover, among the glycolytic enzymes underrepresented in both conditions were GAPDH, ENO1, and PKM, which have been recently demonstrated to promote pro-inflammatory macrophage functions through moonlighting activity (Van den Bossche et al., 2017). A similar downregulation in glycolytic enzymes at early stages of infection has also been observed in *Trypanosoma cruzi*- and HIV-1-infected cells, suggesting a decrease in energy production from glucose at this stage of infection (Ringrose et al., 2008; Li et al., 2016). Interestingly, our transcriptomic data of *R. conorii*-infected THP-1 macrophages also revealed that RRAD (Ras-related glycolysis inhibitor and calcium channel regulator), belongs to one of the most upregulated genes at 1 h post-infection (unpublished results), which further supports the idea of a reduction in host glycolytic activity at an early stage of infection of THP-1 cells with *R. conorii*. Thus, shutdown in host glycolytic and PPP activities early in infection should be further addressed as a possible mechanism of *Rickettsia* to evade macrophage pro-inflammatory responses.

The metabolic characteristics of the TCA cycle and OXPHOS are also distinct between M1 and M2-like phenotypes. An intact TCA cycle and enhanced OXPHOS characterize M2 macrophages, whereas in inflammatory macrophages the TCA cycle has been shown to be broken in two places and OXPHOS impaired (Van den Bossche et al., 2017). These breaks in the TCA cycle, after citrate due to a decrease in expression of isocitrate dehydrogenase 1 (IDH1) and after succinate, lead to accumulation of citrate to meet the biosynthetic demands of inflammatory macrophages, including synthesis of fatty acids, lipids, and prostaglandins, and succinate, an inflammatory signal that stabilizes hypoxia-inducible factor 1 alpha (HIF1α), thereby promoting LPS-induced expression of IL-1α (Van den Bossche et al., 2017). Remarkably, we also observed a reduction in abundance of host IDH1 upon infection with both species, suggestive of a possible impact on citrate accumulation as described for M1-like macrophages. Since succinate dehydrogenase was not quantified in our dataset, it is not possible to infer the presence or absence of the second break in the TCA cycle at this point. In contrast to *R. montanensis*-infected cells, the observed accumulation of several TCA cycle and OXPHOS enzymes in *R. conorii*-infected macrophages differ from the typical hallmarks of the bactericidal M1 phenotype, showing instead higher resemblance of these cells with an M2-like phenotype. Indeed, M2 macrophages obtain much of their energy from fatty acid oxidation and oxidative metabolism, with a massive induction of an oxidative metabolic program, ranging from fatty acid uptake and oxidation to oxidative phosphorylation and mitochondrial respiration (Mills and O'Neill, 2016). Of note, higher accumulation of several fatty acid oxidation enzymes was observed in *R. conorii*-infected cells only, which is again suggestive that this pathogenic SFG *Rickettsia* specifically induces a reprogramming toward an M2-like phenotype. The impact of host cell lipid metabolism during infection has been already studied for several intracellular pathogens (Jordan and Randall, 2017; Shehata et al., 2017). One of the better-documented examples is the ability of dengue virus to promote its replication by inducing lipophagy, a selective autophagy that targets lipid droplets, which further enhances fatty-acid β-oxidation and subsequent viral replication (Jordan and Randall, 2017). We have demonstrated that pharmacological inhibition of FASN activity and reduction of FASN expression, significantly affects the ability of *R. conorii* to replicate within differentiated THP-1 cells. Further analysis of the contributions of *de novo* fatty acid synthesis and fatty acid β-oxidation to rickettsial growth in THP-1 cells and other target cell types is under current investigation.

Furthermore, recent findings suggest that α-ketoglutarate produced via glutaminolysis, which enters the TCA cycle to replenish TCA cycle intermediates, is also an anti-inflammatory metabolite that orchestrates M2 activation of macrophages through different reprogramming processes (Liu et al., 2017). The herein observed increased abundance of the enzymes GOT2 and GLUD1 in *R. conorii*-infected cells, also suggests the possible use of glutamate, which is abundant in cell culture media, to fuel the TCA cycle through conversion into α-ketoglutarate. These results point toward an active anaplerotic flux in response to *R. conorii* infection, which may contribute to balance the levels of TCA intermediates.

Taken together, these data provide further evidence of an M2-like activation program promoted by *R. conorii* during infection of macrophage-like cells. Actually, recent evidence obtained in our laboratory showed that *R. conorii*-infected THP-1 cells (24 h after infection) are unresponsive to a challenge with LPS, which is a known inducer of M1 polarization, while *R. montanensis*-infected cells responded with increased secretion of TNFα (unpublished results). These results are in line with an *R. conorii*-induced reprogramming of THP-1 cells toward an anti-inflammatory phenotype (M2-like).

**Figure 9:**
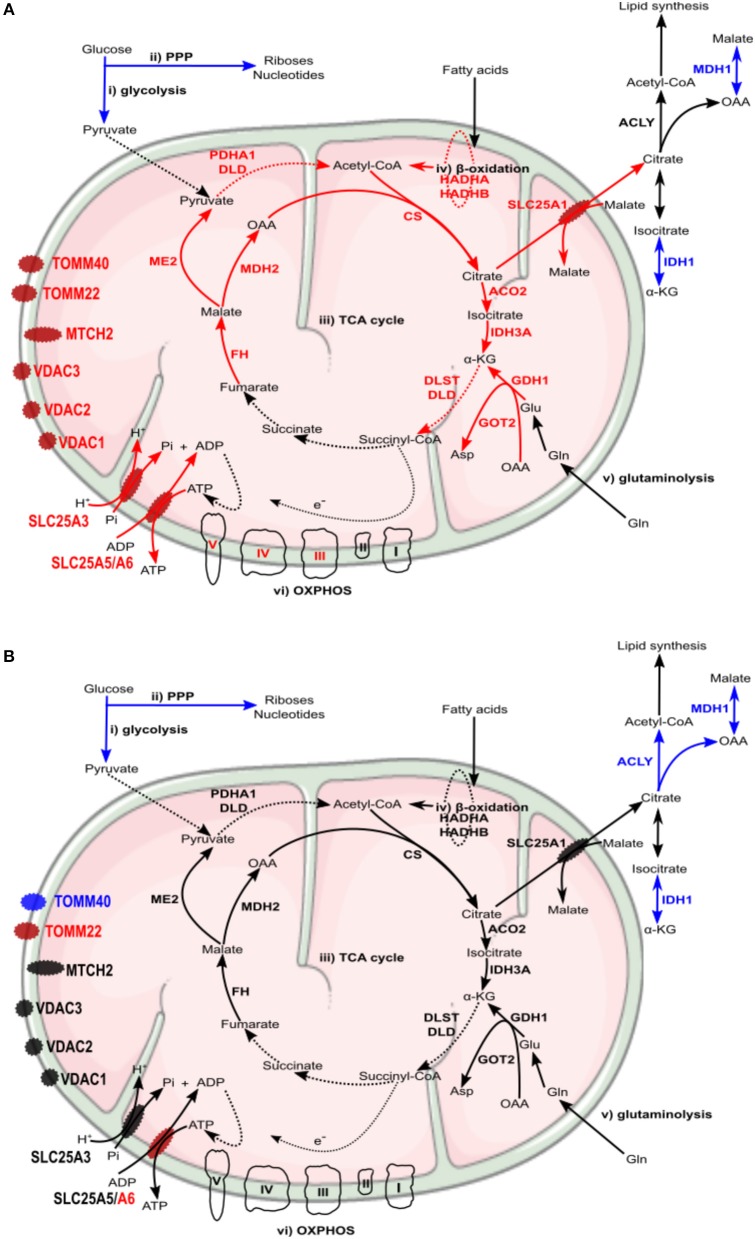
*Rickettsia conorii* and *R. montanensis* trigger a differential metabolic signature in THP-1 macrophages. **(A,B)** Prediction model of alterations in host cell metabolism, based on changes in the abundance of host proteins induced by infection of THP-1 macrophages with *R. conorii* **(A)** or *R. montanensis* **(B)** Increase/decrease in the abundance of host enzymes are predicted to contribute with increase/decrease in activity for the respective biological enzymatic activity and are represented in red and blue, respectively. Enzymes quantified in our analysis but with no alteration in abundance upon infection are represented in black **(A)** In *R. conorii*-infected THP-1 macrophages, glycolysis (i) and pentose phosphate pathway (ii) are predicted to be reduced at 24 h post-infection. This may impact pyruvate production from glycolysis as well as production of riboses, nucleotides and ROS from PPP, globally contributing to reduce pro-inflammatory signals. Several TCA cycle enzymes (iii) were found overrepresented upon infection, suggesting an increase in TCA cycle activity. Acetyl-CoA production from fatty-acid β-oxidation (iv) and glutamine anaplerosis (v) may contribute to replenish the TCA cycle which may result in a sustained ATP production via oxidative phosphorylation (vi). Accumulation of several inner and outer membrane transporters is suggestive of a metabolic configuration with higher needs in metabolic supply and demand. **(B)** In *R. montanensis*-infected THP-1 macrophages, pyruvate production from glycolysis (i) is also predicted to be reduced at this time of infection. However, in contrast with *R. conorii*, unchanged levels of enzymes of the TCA cycle (iii), fatty-acid β-oxidation (iv), glutaminolysis (v), and proteins from the respiratory complex (vi) found in *R. montanensis*-infected cells, together with no alterations observed in most of the quantified mitochondrial transporters, suggests very distinct metabolic requirements between infection conditions (see Tables 3, 4 for details).

Recent studies with different intracellular pathogens have indeed demonstrated that the metabolic conditions of M2-like macrophages represent a more favorable replication niche than the inflammatory M1 phenotype (Price and Vance, 2014; Eisenreich et al., 2017). In line with these observations, our results suggest that differences in host cell metabolism promoted by infection with each rickettsial species may indeed reflect differential macrophage activation modes that either favor (*R. conorii*) or restrict (*R. montanensis*) intracellular bacterial proliferation. A summary of the main differences in protein content of metabolic pathway components observed between infection conditions, and potential impact on metabolic fluxes, are illustrated in Figures 9A,B. Overall, our results with *R. conorii* point toward a shift away from glycolysis but with an apparently higher metabolic flux directed to the TCA cycle through other metabolic pathways, such as fatty acid oxidation and glutaminolysis, that are known to fuel this cycle. This might be used to increase TCA cycle activity augmenting the levels of NADH, GTP, and FADH_2_, which could be further used in cellular respiration steps to produce ATP, and/or to replenish the TCA cycle to compensate for diversion of metabolites (e.g., citrate) to other metabolic pathways. Among mitochondrial transporters found in higher abundance in *R. conorii*-infected cells were SLC25A3 and SCL25A5/SLC25A which are important for ADP phosphorylation and ADP/ATP translocation between the mitochondria and the cytosol. SLC25A1, which transports citrate out of the mitochondria, and VDAC1, VDAC2, and VDAC3 that control the flux of various metabolites and ions through the mitochondrial outer membrane, were also found in higher abundance in *R. conorii*-infected cells.

These metabolic adaptations may not only help to counteract host defense mechanisms, promoting a more comfortable replication niche but may also reflect the complex interconnection with the metabolic and energetic requirements of *R. conorii* itself. Reductive genome evolution has resulted in the loss of many metabolic pathways, which culminates with *Rickettsia* species being strictly dependent on host metabolites to survive and proliferate (Darby et al., 2007). *Rickettsia* display a limited oxidative metabolism. Both glycolysis/gluconeogenesis and PPP enzymatic activities are undetectable although there is evidence of a functional pyruvate dehydrogenase complex and TCA cycle (Coolbaugh et al., 1976; Phibbs and Winkler, 1982; Winkler and Daugherty, 1986; Renesto et al., 2005; Driscoll et al., 2017). Together with other cofactors, several metabolites have been shown (glutamate, glutamine, serine, glycine) and predicted (malate, pyruvate, α-ketoglutarate) to be imported from the host to fuel the TCA cycle, which in turn feeds the pathways responsible for peptidoglycan synthesis and for ATP generation (OXPHOS) (Driscoll et al., 2017). Interestingly, the TCA-cycle intermediate aspartate, which is essential for biosynthesis of peptidoglycan precursors, is also predicted to be imported by *R. conorii* cells. In addition, *Rickettsia* is also able to import ATP from the host via an ATP/ADP symporter, and it has been suggested that this dual mechanism for energy supply may reflect the adaptation of *Rickettsia* to the metabolic activity of the host cell (Eisenreich et al., 2013; Driscoll et al., 2017). Remarkably, the *R. conorii*-induced host metabolic configuration anticipated in this work (Figure 9A) appear to favor the generation of several metabolites also required by *Rickettsia*, such as glutamate, pyruvate, malate, α-ketoglutarate, and aspartate. Moreover, fueling the TCA cycle through fatty acid oxidation can allow the production of very high amounts of ATP by the host, which may also be imported by *R. conorii*. Therefore, promoting the adaptation of host metabolic pathways toward the generation of the carbon substrates and energy required for bacterial proliferation and for sustaining host survival might be one of the strategies used by *R. conorii* to reduce the metabolic burden put on the host cell by an intracellular pathogen so heavily dependent on host metabolism (Driscoll et al., 2017). Curiously, mitochondrial porins (VDAC), which have been hypothesized to be hijacked by *Rickettsia* to be used as transport systems (Emelyanov and Vyssokikh, 2006; Emelyanov, 2009), were found in higher abundance in *R. conorii*-infected cells, urging for further studies to explore putative porin incorporation in rickettsial cells during infection. Understanding rickettsial-host (macrophage) metabolic interconnections and which/how bacterial effectors and transporters control these complex adaptation processes emerge has an exciting area for future research.

By maintaining the levels of many regulatory proteins and removing damaged or misfolded proteins, the UPS is involved in a variety of cellular processes, including quality control of the proteome, antigen presentation or stress responses (Vilchez et al., 2014; Bentea et al., 2017). Bacterial and viral pathogens have evolved various strategies to exploit the UPS depending on their needs (Randow and Lehner, 2009; Zhou and Zhu, 2015), and it is now known that several bacterial effectors can inhibit specific UPS steps to modulate host cell immune responses and bacterial clearance (Kim et al., 2014). However, in the case of bacterial pathogens, this modulation has been mainly associated with interference of ubiquitination/deubiquitination steps by different strategies (Kim et al., 2014). Strikingly, our results show that infection of THP-1 macrophages with both *R. conorii* and *R. montanensis* have a dramatic impact on the proteasome, with several of the subunits forming this proteolytic machinery found in reduced abundance in infected cells. Among affected subunits, we found several elements of the 20S core particle, including subunits β1 and β2 which display caspase-like and trypsin-like proteolytic activities (Vilchez et al., 2014), as well as both subunits of the proteasome activator complex PA28, important for assembly of the immunoproteasome (McCarthy and Weinberg, 2015). Moreover, several subunits of the 19S regulatory cap were also significantly underrepresented in the *R. montanensis* dataset. The decreased abundance of all these components suggests an impairment of proteasome activity at this time post-infection. To our knowledge, this is the first time the proteasome itself is shown to be affected as a result of a bacterial infection. This raises exciting questions regarding how and why *Rickettsia* modulate host proteasome function since this likely interferes with different cellular processes. Interestingly, many viruses have mechanisms of interfering with proteasome function by preventing transcriptional upregulation or by direct interaction of viral proteins with immunoproteasome subunits (McCarthy and Weinberg, 2015). In these viral infections, downregulation of immunoproteasome activity has been suggested as a mechanism to reduce the generation of viral peptide antigens to be presented on the MHC class I complex, and thereby avoid host immune surveillance (McCarthy and Weinberg, 2015). Rickettsial antigens can be presented by both MHC class I and MHC class II pathways (Fang et al., 2007; Osterloh, 2017). The herein observed effect on different proteasome and proteasome activator (PA28) subunits, raise the exciting possibility that rickettsial species may also exploit this proteolytic machinery as a sophisticated strategy to decrease antigen peptide generation, decreasing/inhibiting antigen presentation in macrophages. Therefore, the molecular mechanisms underlying this interference with the proteasome and its impact on the regulation of immune responses and the possible contribution to rickettsial evasion of immune surveillance should be further investigated. As mentioned, impairment of proteasome function may have other implications (Ferrington and Gregerson, 2012; Kaur and Batra, 2016; Bentea et al., 2017). It has been demonstrated that pharmacologic inhibition of the proteasome in macrophages leads to a dysregulation in inflammatory signaling, resulting in a conversion to an anti-inflammatory phenotype (Cuschieri et al., 2004). Although the interference with proteasome components appears to be a response induced by both rickettsial species, regardless of pathogenicity, we cannot exclude the potential impact of proteasome dysfunction on the production of inflammatory mediators. Combined with the observed changes in cellular metabolism, the modulation of proteasome function may also positively contribute to establish a more favorable niche for *R. conorii* survival.

**Figure 10:**
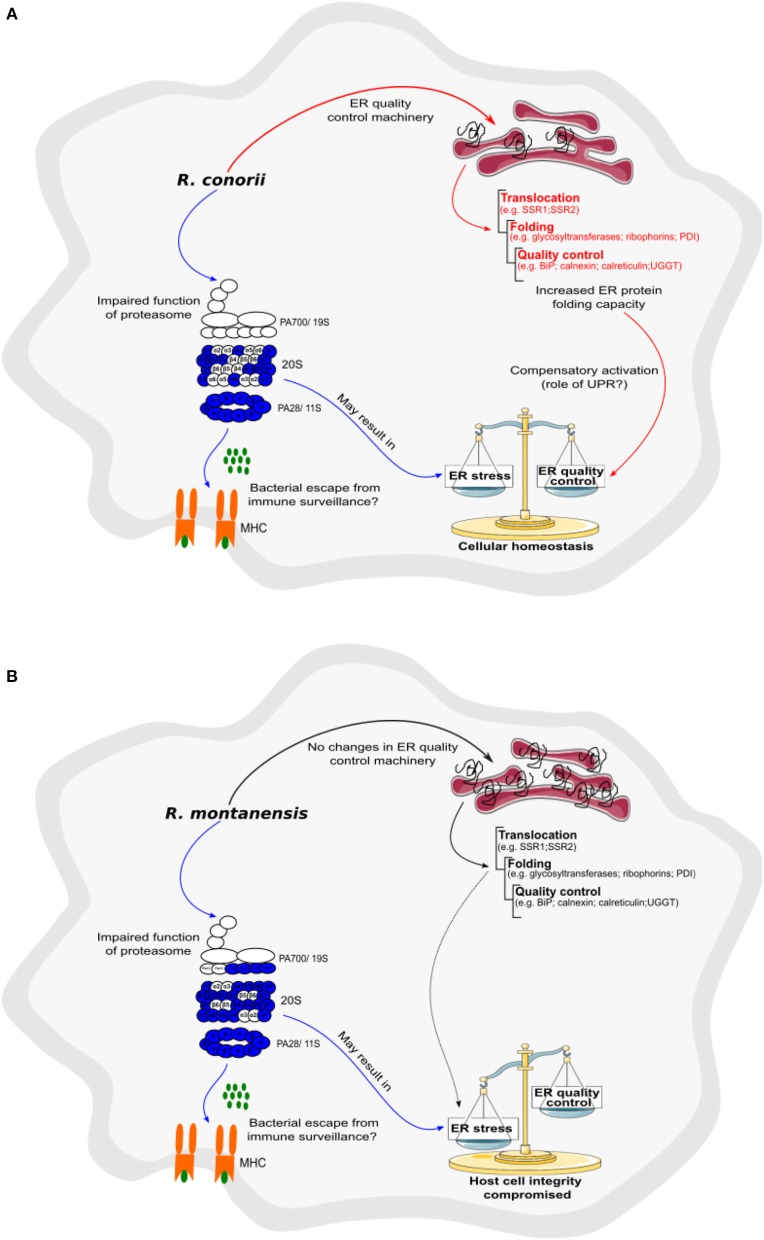
*Rickettsia conorii*, but not *R. montanensis*, may be able to restore host cell homeostasis by increasing ER folding capacity. **(A,B)** Prediction model of alterations in proteasome and protein processing in endoplasmic reticulum activity, based on changes in the abundance of host proteins, that are induced by the infection of THP-1 macrophages with *R. conorii* **(A)** or *R. montanensis* **(B)**. Increase/decrease in the abundance of host enzymes are predicted to contribute with increase/decrease in activity and are represented in red and blue, respectively. Increase, no alteration, or decrease in the abundance of host enzymes are predicted to contribute with increase, unchanged or decrease activity for the respective biological function and are represented in red, black, and blue arrows, respectively. **(A)** In *R. conorii*-infected THP-1 macrophages, several proteasome and immunoproteasome activator subunits are underrepresented at 24 h post-infection, which may lead to a decrease in antigen peptide generation, and subsequent decrease in antigen presentation by MHC complex Type I, and bacterial evasion from immune system surveillance. Decrease in proteasome activity may lead to an accumulation of misfolded proteins in ER, inducing ER stress. However, *R. conorii* specifically increases the abundance of several ER proteins involved protein translocation, folding, and quality control, which may be a compensatory mechanism activated by the UPR. **(B)** In *R. montanensis*-infected THP-1 macrophages, several proteasome and immunoproteasome activator subunits are also underrepresented at 24 h post-infection, which may lead to a decrease in antigen peptide generation (ii), and subsequent antigen presentation by MHC class I and bacteria evasion from immune system surveillance. Decrease in proteasome activity may lead to an accumulation of misfolded proteins and induction of ER stress. However, in contrast with *R. conorii, R. montanensis* infection did not result in increased levels of ER quality control components, likely resulting in the inability of *R. montanensis* to restore host cell homeostasis (see Table 5 for details).

An impact in proteasome function is also likely to induce ER stress through the accumulation of unfolded proteins (Thibaudeau et al., 2018; VerPlank et al., 2018). Moreover, a wide range of other cellular perturbations induced by infection, such as nutrient depletion, disruption of the secretory pathway, the accumulation of ROS or increase of free fatty acids, may also result in perturbations in ER homeostasis (Galluzzi et al., 2017). Another noticeable difference between *R. conorii* and *R. montanensis*-infected macrophages was the observed *R. conorii*-specific accumulation of various components of the ER quality control machinery. Among these proteins are those associated with translocation across the ER membrane, as well as several proteins related to protein folding and quality control check, including several glycosyltransferases, disulfide isomerases, classical, and non-classical chaperones, as well as glycan modification enzymes. These results are suggestive of a host response to counteract ER stress, which appears to be specifically triggered by the pathogenic *Rickettsia* only. Indeed, increasing the ER protein folding capacity has been shown as one of the compensatory mechanisms of the UPR to re-establish ER homeostasis (Janssens et al., 2014; Martins et al., 2016; Hetz and Papa, 2018). Whether this impact in ER-quality control machinery and in the UPR as a whole is being actively manipulated by *R. conorii* or is an indirect response to other cellular effects remains to be elucidated. However, the differential accumulation patterns in ER proteins observed in *R. conorii* and *R. montanensis* infection datasets, strongly suggests that *R. conorii* may have the ability to manipulate ER stress signaling to its benefit, as demonstrated for other successful intracellular pathogens (Celli and Tsolis, 2015; Galluzzi et al., 2017). Main differences in proteasome and ER protein abundance observed in this work, and possible responses associated with these changes, are summarized in Figures 10A,B. A reduced representation of proteasome subunits, found under both infection conditions, may interfere with *Rickettsia* antigen presentation by MHC class I and recognition by the immune system. However, this possible impairment of proteasome function may trigger ER stress through an accumulation of misfolded proteins in the lumen of the ER. While *R. montanensis* promoted no significant changes in host ER proteins, *R. conorii* induced a positive modulation of the ER folding capacity, likely contributing to re-establish ER homeostasis. This may help to restore cellular homeostasis and maintain host cell integrity for pathogen replication. Therefore, the ability to restore ER homeostasis may be another critical feature to help pathogenic rickettsial species like *R. conorii* successfully establish infections within phagocytic cells.

The proteomic profiles herein presented contribute significant insights toward a more in-depth understanding on the modulation of THP-1 macrophage responses upon infection with two rickettsial species that show different intracellular fates in these cells (Curto et al., 2016). Our results revealed a substantial metabolic reprogramming as well as a modulation of ER folding capacity, specifically induced by the pathogen *R. conorii*. Globally, this helps to unfold the intricate pattern of modulation triggered by a pathogenic *Rickettsia* to control macrophage homeostasis and to maintain a viable intracellular niche. By illuminating the still very poorly studied aspects of macrophage-*Rickettsia* interactions, such as metabolic adaptations, the UPR or proteasome dysfunction, our work provides an important framework for future investigations that are likely to lead to an improved understanding of the link between these mechanisms and rickettsial pathogenicity.

## Data Availability

The mass spectrometry proteomics data have been deposited to the ProteomeXchange Consortium via the PRIDE (Vizcaino et al., 2016) partner repository, with the identifier PXD010330.

## Author Contributions

PC, IS, and JM: conceptualization; PC, CS, PA, and IS: investigation; PC, CS, PA, and BM: formal analysis; PC, CS, and IS: writing original draft; PC, CS, PA, BM, IS, and JM: writing review and editing; IS and JM: supervision; BM and JM: resources; IS, BM, and JM: funding acquisition.

### Conflict of Interest Statement

The authors declare that the research was conducted in the absence of any commercial or financial relationships that could be construed as a potential conflict of interest.
