# Supplementary material for: A Pathogen and a Non-pathogen Spotted Fever Group Rickettsia Trigger Differential Proteome Signatures in Macrophages
Source: Front Cell Infect Microbiol. 2019 Mar 6;9:43. doi: 10.3389/fcimb.2019.00043 (PMC6414445; doi:10.3389/fcimb.2019.00043)
Supplement: Supplementary file 1 [file Table_1.DOCX]

**Supplementary Table 1.** Information about the windows used in SWATH acquisition (cont. next pages).

| Window | Start Mass (Da) | Stop Mass (Da) | Mass Interval (Da) | CES |
| --- | --- | --- | --- | --- |
| Window 1 | 349.5 | 360.9 | 11.4 | 5 |
| Window 2 | 359.9 | 375.2 | 15.3 | 5 |
| Window 3 | 374.2 | 389.2 | 15 | 5 |
| Window 4 | 388.2 | 402.2 | 14 | 5 |
| Window 5 | 401.2 | 415.3 | 14.1 | 5 |
| Window 6 | 414.3 | 427.4 | 13.1 | 5 |
| Window 7 | 426.4 | 439.1 | 12.7 | 5 |
| Window 8 | 438.1 | 449.9 | 11.8 | 5 |
| Window 9 | 448.9 | 460.7 | 11.8 | 5 |
| Window 10 | 459.7 | 471.1 | 11.4 | 5 |
| Window 11 | 470.1 | 480.5 | 10.4 | 5 |
| Window 12 | 479.5 | 490 | 10.5 | 5 |
| Window 13 | 489 | 499 | 10 | 5 |
| Window 14 | 498 | 508 | 10 | 5 |
| Window 15 | 507 | 516.5 | 9.5 | 5 |
| Window 16 | 515.5 | 525.1 | 9.6 | 5 |
| Window 17 | 524.1 | 533.2 | 9.1 | 5 |
| Window 18 | 532.2 | 540.8 | 8.6 | 5 |
| Window 19 | 539.8 | 548.5 | 8.7 | 5 |
| Window 20 | 547.5 | 555.7 | 8.2 | 5 |
| Window 21 | 554.7 | 563.4 | 8.7 | 5 |
| Window 22 | 562.4 | 570.6 | 8.2 | 5 |
| Window 23 | 569.6 | 577.8 | 8.2 | 5 |
| Window 24 | 576.8 | 585.4 | 8.6 | 5 |
| Window 25 | 584.4 | 592.6 | 8.2 | 5 |
| Window 26 | 591.6 | 600.3 | 8.7 | 5 |
| Window 27 | 599.3 | 607.9 | 8.6 | 5 |
| Window 28 | 606.9 | 615.6 | 8.7 | 5 |
| Window 29 | 614.6 | 623.2 | 8.6 | 5 |
| Window 30 | 622.2 | 630.9 | 8.7 | 5 |
| Window 31 | 629.9 | 638.5 | 8.6 | 5 |
| Window 32 | 637.5 | 646.2 | 8.7 | 5 |
| Window 33 | 645.2 | 653.8 | 8.6 | 5 |
| Window 34 | 652.8 | 661.5 | 8.7 | 5 |
| Window 35 | 660.5 | 669.1 | 8.6 | 5 |
| Window 36 | 668.1 | 677.2 | 9.1 | 5 |
| Window 37 | 676.2 | 685.3 | 9.1 | 5 |
| Window 38 | 684.3 | 693.9 | 9.6 | 5 |
| Window 39 | 692.9 | 702.9 | 10 | 5 |
| Window 40 | 701.9 | 711.9 | 10 | 5 |
| Window 41 | 710.9 | 721.3 | 10.4 | 5 |
| Window 42 | 720.3 | 731.2 | 10.9 | 5 |
| Window 43 | 730.2 | 741.6 | 11.4 | 5 |
| Window 44 | 740.6 | 752.4 | 11.8 | 5 |
| Window 45 | 751.4 | 763.6 | 12.2 | 5 |
| Window 46 | 762.6 | 775.8 | 13.2 | 5 |
| Window 47 | 774.8 | 787.9 | 13.1 | 5 |
| Window 48 | 786.9 | 800.5 | 13.6 | 8 |
| Window 49 | 799.5 | 814.5 | 15 | 8 |
| Window 50 | 813.5 | 829.3 | 15.8 | 8 |
| Window 51 | 828.3 | 845.5 | 17.2 | 8 |
| Window 52 | 844.5 | 865.3 | 20.8 | 8 |
| Window 53 | 864.3 | 886.5 | 22.2 | 8 |
| Window 54 | 885.5 | 911.2 | 25.7 | 8 |
| Window 55 | 910.2 | 939.1 | 28.9 | 8 |
| Window 56 | 938.1 | 972 | 33.9 | 8 |
| Window 57 | 971 | 1008.4 | 37.4 | 10 |
| Window 58 | 1007.4 | 1053.4 | 46 | 10 |
| Window 59 | 1052.4 | 1120 | 67.6 | 10 |
| Window 60 | 1119 | 1249.6 | 130.6 | 10 |
