# Supplementary material for: A Pathogen and a Non-pathogen Spotted Fever Group Rickettsia Trigger Differential Proteome Signatures in Macrophages
Source: Front Cell Infect Microbiol. 2019 Mar 6;9:43. doi: 10.3389/fcimb.2019.00043 (PMC6414445; doi:10.3389/fcimb.2019.00043)
Supplement: Supplementary file 4 [file Data_Sheet_1.PDF]

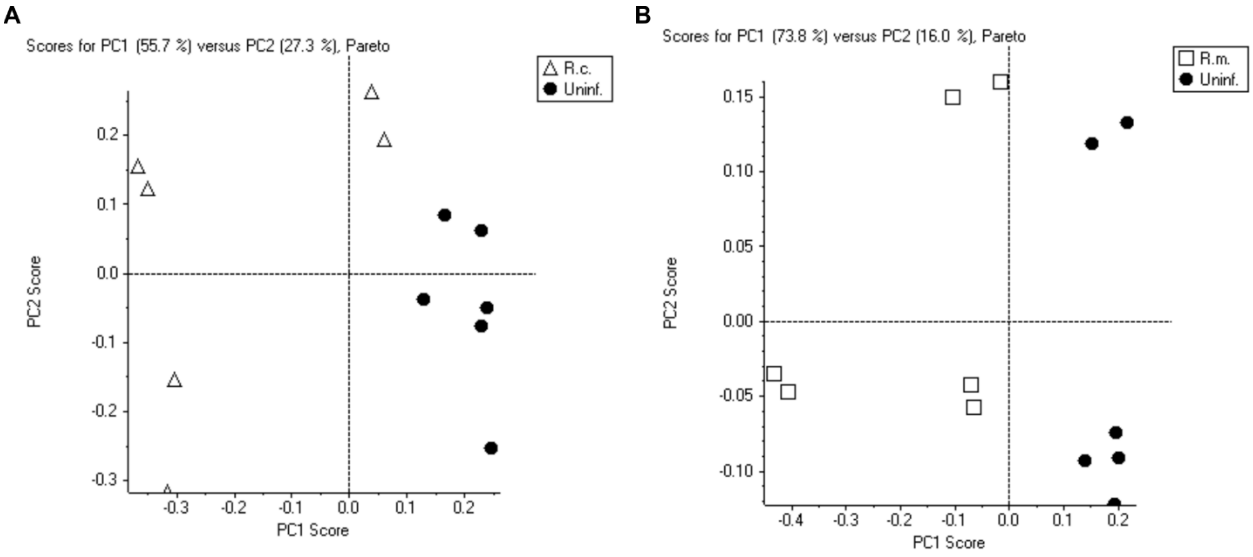

Supplementary Figure 1. Principal component analysis (PCA) plots of global changes in proteome profiles. (A-B) PCA plot was performed by importing the quantification data of all proteins considered as altered for *R. conorii*-infected THP-1 macrophages *vs.* uninfected cells **(A)** and for *R. montanensis*-infected THP-1 macrophages *vs.* uninfected cells **(B)**. PCA was performed using the software MarkerView (v1.2.1, Sciex) and the axes show the first two principal components, with the fraction of explained variance in the parenthesis.
