# Supplementary material for: A Pathogen and a Non-pathogen Spotted Fever Group Rickettsia Trigger Differential Proteome Signatures in Macrophages
Source: Front Cell Infect Microbiol. 2019 Mar 6;9:43. doi: 10.3389/fcimb.2019.00043 (PMC6414445; doi:10.3389/fcimb.2019.00043)
Supplement: Supplementary file 5 [file Data_Sheet_2.PDF]

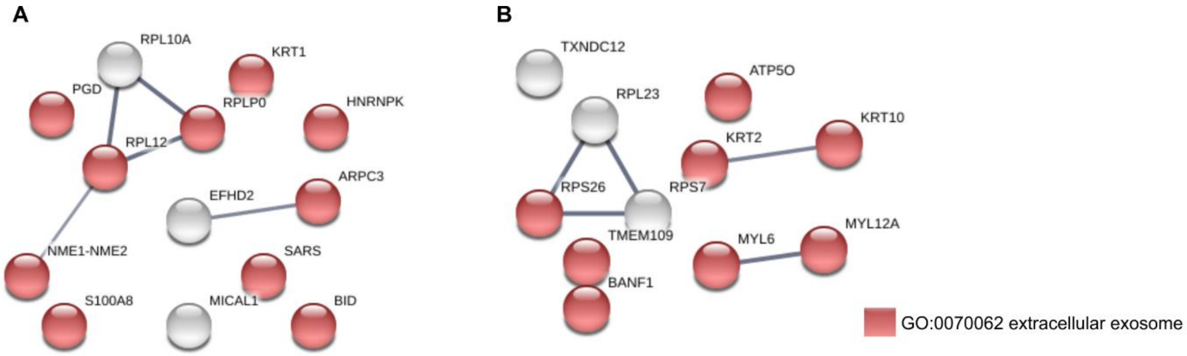

Supplementary Figure 2. Protein-protein interaction networks of host proteins with altered abundance in one infection condition. (A-B) Protein-protein interaction network for the 13 host proteins with decreased abundance in *R. conorii*-infected THP-1 macrophages but unchanged levels in *R. montanensis*-infected cells **(A)** and the 11 host proteins with increased abundance in *R. montanensis*-infected THP-1 macrophages but unchanged levels in *R. conorii*-infected cells **(B)**. List of the individual host proteins for each independent analysis can be found in S2 Table. The analysis was carried out with STRING 10.5 (http://string-db.org/) using high confidence (0.7) score. Nodes are represented with different colors according to their categorization in gene ontology (GO) terms.
