# Supplementary material for: A Pathogen and a Non-pathogen Spotted Fever Group Rickettsia Trigger Differential Proteome Signatures in Macrophages
Source: Front Cell Infect Microbiol. 2019 Mar 6;9:43. doi: 10.3389/fcimb.2019.00043 (PMC6414445; doi:10.3389/fcimb.2019.00043)
Supplement: Supplementary file 6 [file Data_Sheet_3.PDF]

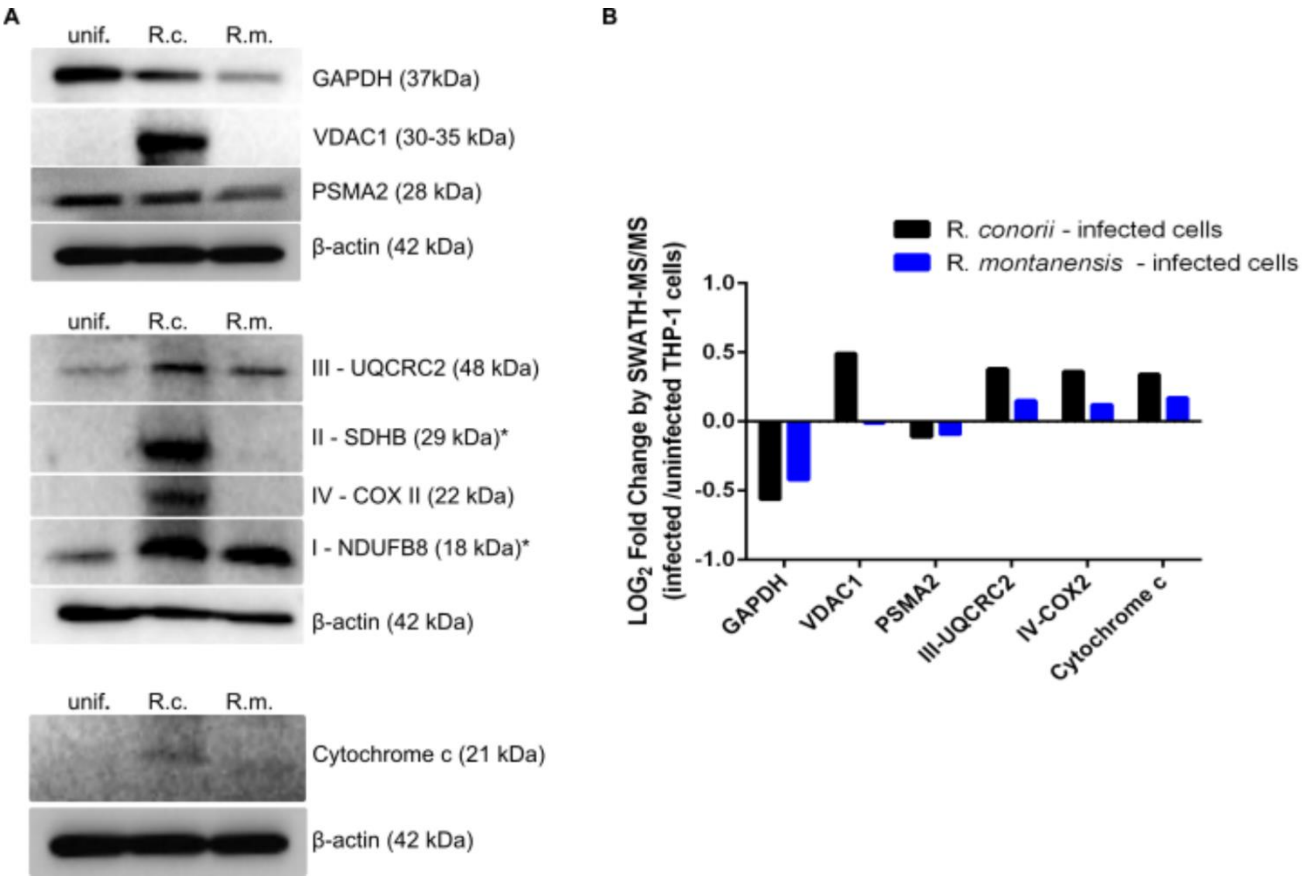

Supplementary Figure 3. Changes in the abundance of several host proteins involved in metabolic processes were validated by Western blot analysis. (A) Western blot analysis of total protein extracts (10 μg of total protein) from uninfected THP-1 macrophages (uninf.), R. conorii-infected THP-1 macrophages (R.c.), and R. montanensis-infected THP-1 macrophages (R.m.) for several host proteins. Protein samples were probed for glyceraldehyde-3-phospate dehydrogenase (GAPDH, P04406) (glycolysis); voltage dependent anion channel 1 (VDAC1, P21796) (mitochondrial transporter); proteasome subunit alpha type-2 (PSMA2, P25787) (proteasome); ubiquinol-cytochrome c reductase core protein II (UQCRC2, P22695) (OXPHOS, complex III); succinate dehydrogenase iron-sulfur subunit (SDHB, P21912) (OXPHOS, complex II); cytochrome c oxidase subunit II (COX2, P00403) (OXPHOS, complex IV); NADH dehydrogenase 1 beta subcomplex subunit 8 (NDUFB8, O95169) (OXPHOS, complex I); and cytochrome c 1 (CYC1, P08574) (OXPHOS). Immunoblot analysis with anti-β-actin was used as protein loading control and PSMA2 was used as a control protein with no significant quantitative changes by SWATH-MS. Asterisk (*) means that the protein was not confidentially quantified by SWATH-MS. **(B)** Bar chart of $\log_2$ fold change for each individual protein as determined by SWATH-MS between R. conorii- and R. montanensis-infected and uninfected THP-1 macrophages for comparison.
